# Supplementary material for: Synthesis, Antimicrobial and Mutagenic Activity of a New Class of d-Xylopyranosides
Source: Antibiotics (Basel). 2023 May 10;12(5):888. doi: 10.3390/antibiotics12050888 (PMC10215737; doi:10.3390/antibiotics12050888)
Supplement: Supplementary file 1 [file antibiotics-12-00888-s001.zip › antibiotics-2379650-supplementary.pdf]

antibiotics

MDPI

Supplementary Materials

Synthesis, Antimicrobial and Mutagenic Activity of a New Class of D-Xylopyranosides

Karol Sikora [1,2], Piotr Szweda [3], Karolina Słoczyńska [4], Justyna Samaszko-Fiertek [1], Janusz Madaj [1], Beata Liberek [1], Elżbieta Pękala [4] and Barbara Dmochowska [1,*]

[1] Faculty of Chemistry, University of Gdańsk, Wita Stwosza 63, 80-308 Gdańsk, Poland; karol.sikora@gumed.edu.pl (K.S.); j.samaszko-fiertek@ug.edu.pl (J.S.-F.); janusz.madaj@ug.edu.pl (J.M.); beata.liberek@ug.edu.pl (B.L.)
[2] Department of Inorganic Chemistry, Faculty of Pharmacy, Medical University of Gdańsk, Al. Gen. J. Hallera 107, 80-416 Gdańsk, Poland
[3] Department of Pharmaceutical Technology and Biochemistry, Faculty of Chemistry, Gdańsk University of Technology, G. Narutowicza 11/12, 80-233 Gdańsk, Poland; piotr.szweda@pg.edu.pl
[4] Department of Pharmaceutical Biochemistry, Faculty of Pharmacy, Jagiellonian University Medical College, Medyczna 9, 30-688 Krakow, Poland; karolina.sloczynska@uj.edu.pl (K.S.); elzbieta.pekala@uj.edu.pl (E.P.)
* Correspondence: basia.dmochowska@ug.edu.pl

1. General methods

Commercial xylose (Fluka) was used. All reactions were monitored by a thin-layer chromatography (TLC), using pre-coated silica gel 60 F_{254} plates (100–200 mesh). Detection of spots was carried out by spraying 5% solution of H_2SO_4 in methanol and charring. Purification of products was performed using FLASH (Interchim PuriFlash 450) chromatography, 30 µm silica gel as a stationary phase. The structures of all products were determined with the ^{1}H, ^{13}C, and 2D (HSQC, COSY) NMR, Bruker AVANCE III, at 400 or 500 MHz and 100 or 125 MHz for ^{1}H, ^{13}C, respectively, in $CDCl_3$ or D_2O as solvents. Mass spectrometry analysis was done using Bruker Biflex III MALDI-TOF-MS spectrometer (with 2,5-dihydroxybenozoic acid (DHB) matrix) and HRMS was performed using electrospray ionization with time-of-flight mass analysis (Agilent Technologies 6550 iFunnel Q-TOF). Optical rotation was measured with 343 PerkinElmer polarimeter.

Table S1. Reaction yields and solvents used for the quaternization reaction at 70 °C.

No.	Substrate	Amine	Solvent	Product	Yield [%]
1	**2**	pyridine	-	**4a**	67
2		trimethylamine	ethanol	**4b**	87
3		*N,N*-dimethylhexylamine	acetonitrile	**4c**	72
4		*N,N*-dimethyloctylamine	acetonitrile	**4d**	70
5	**3**	pyridine	-	**5a**	75
6		trimethylamine	ethanol	**5b**	55
7		*N,N*-dimethylhexylamine	acetonitrile	**5c**	68
8		*N,N*-dimethyloctylamine	acetonitrile	**5d**	70

2. Antimicrobial Activity

The preliminary assessment of antimicrobial potential of the synthesized compounds was performed against two reference strains of bacteria: *Staphylococcus aureus* ATCC 25923 and *Escherichia coli* K12 and also two reference strains of pathogenic yeasts, *Candida albicans* ATCC10231 and *Candida glabrata* DSMZ11226. Moreover, 20 clinical isolates of *S. aureus* collected from patients with skin and soft tissue infections (n = 8) and derived from subclinical bovine mastitis milk samples (n = 12) were used for evaluation of anti-staphylococcal activity of two compounds that were found to be most promising in preliminary

test. Four of these strains were classified as MRSA (Methicillin-Resistant *Staphylococcus aureus*); they were mecA positive and did not exhibit susceptibility to methicillin. The MICs (Minimum Inhibitory Concentration) values for all compound were determined by CLSI (Clinical and Laboratory Standards Institute) recommended microdilution methods with minor modifications [1,2]. Briefly, the 8.192 mg/mL (8192 µg/mL) solutions of the synthesized compounds were prepared in Mueller-Hinton Broth 2 - cation adjusted (MHB2) for evaluation of antibacterial activity and in RPMI medium in the case of evaluation of the antifungal activity. Subsequently, the serial, two-fold dilutions of the tested agents (over the range of concentrations from 8192 to 16 µg/mL) were prepared in 96-well microtitration plates in the final volume of 100 µL of the appropriate medium. The pure bacterial cultures of (both the reference and clinical strains) were grown on the Mueller-Hinton Agar (MHA) for 18–24 h at 37 °C and yeasts strains were cultivated on Sabouraud Dextrose Agar (SDA) in the same conditions. A loop of a pure culture of each strain tested (three to five colonies separately growing on agar medium) was suspended in the sterile PBS (phosphate buffered saline, pH 7.4 at 25 °C, purchased from Sigma) solution to get an optical density OD_{600} = 0.13 (for bacteria - equal to the cells concentration approximately 1 x 10^8 CFU/mL) or OD_{660} = 0.1 (for yeasts – equal to the cells concentration approximately 1 x 10^6 CFU/mL). The obtained suspensions of the cells were next diluted 1:100 (*v*/*v*) in the MHB2 medium (bacteria) and 1:50 in RPMI medium (yeasts). One hundred µL of the cells' suspension was finally loaded into the wells of plates prepared in advance, which contained 100 µL of two-fold dilutions of the tested agents. The positive growth control of each strain (both bacteria and yeasts) was performed in the wells without the tested substances. The negative control containing only the media was included in each assay. The plates were incubated for 24 h at 37 °C. Following the incubation period, determination of the MIC values of the tested agents was carried out by measuring the absorbance at 531 nm using a Victor3 microplate reader (Perkin Elmer, Inc., Waltham, MA, USA). The lowest concentration of the agent causing inhibition of growth equal to or higher than 90% (MIC_{90}) of growth control was taken as the MIC value. Each test was repeated three times.

3. Mutagenic Assay—the Ames Test

Salmonella typhimurium TA98 strain was kindly given by Dr. T. Nohmi, Division of Genetics and Mutagenesis, National Institute of Hygienic Sciences, Tokyo, Japan. *Salmonella* cultures were prepared by inoculation form stock cultures into nutrient broth No. 2 (Oxoid, Hampshire, UK) and incubation for 12 h at 37 °C. Next, 100 µL of the bacterial culture (approximately 1-2 × 10^8 bacteria/ tube), 50 µL of the investigated compound at different concentrations (final concentrations used in the study were 2000, 500, 100, 20 and 4 µg/plate, diluted in DMSO), and 500 µL of a buffer solution (0.1 mM, pH=7.4) were mixed and preincubated for 30 min at 37 °C. Then, 2 mL of the top agar containing *histidine/biotin solution* were added to the incubation mixture. The mixture was then poured onto the surface of glucose minimal agar plates. After 48 h revertant colonies were counted. Negative (DMSO) and positive (*4-Nitro-o-phenylenediamine*, 4-NPD, 10 µg/plate, Sigma-Aldrich, St Louis, MO, USA) controls were performed in parallel in each experiment. Experiments were performed in triplicate [3,4]. The mutagenic index (MI) was calculated for each concentration tested, this being the average number of revertants per plate with the test compound divided by the average number of revertants per plate with the negative (solvent) control. A sample was considered mutagenic when a dose–response relationship was detected and at least 2-fold increase in the number of mutants (MI ≥ 2) was observed with at least one concentration [5,6].

2-Bromoethyl 2′,3′,4′-tri-O-acetyl-β-D-xylopyranoside (**2**)

^{1}H NMR (400 MHz, $CDCl_3$):

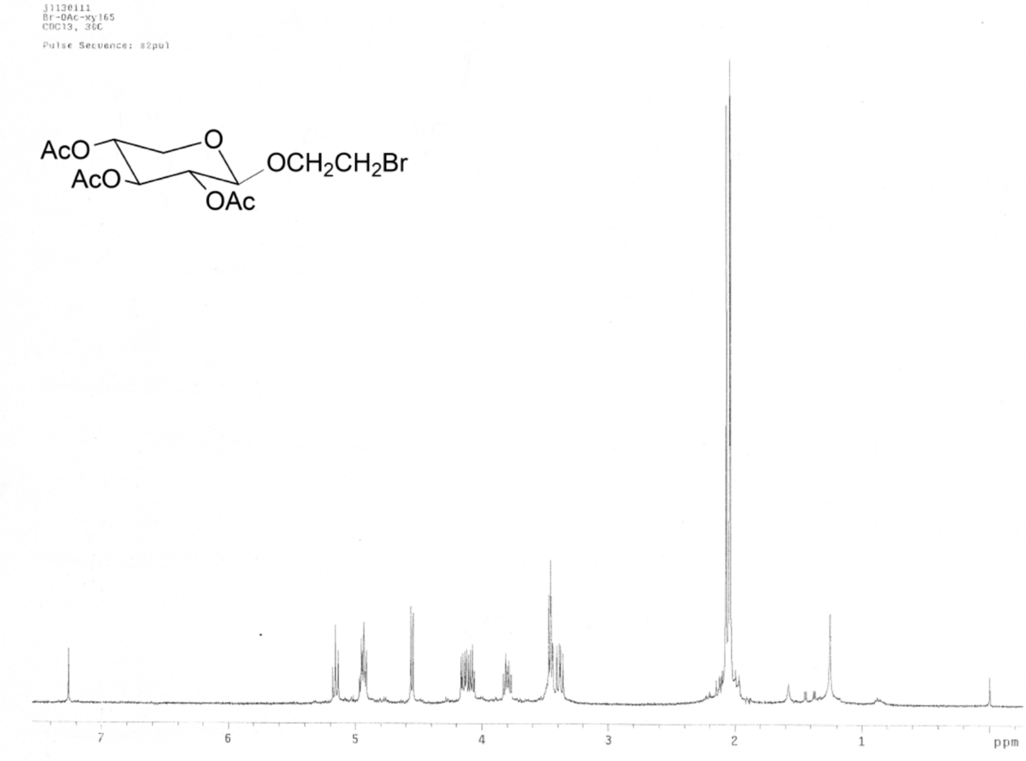

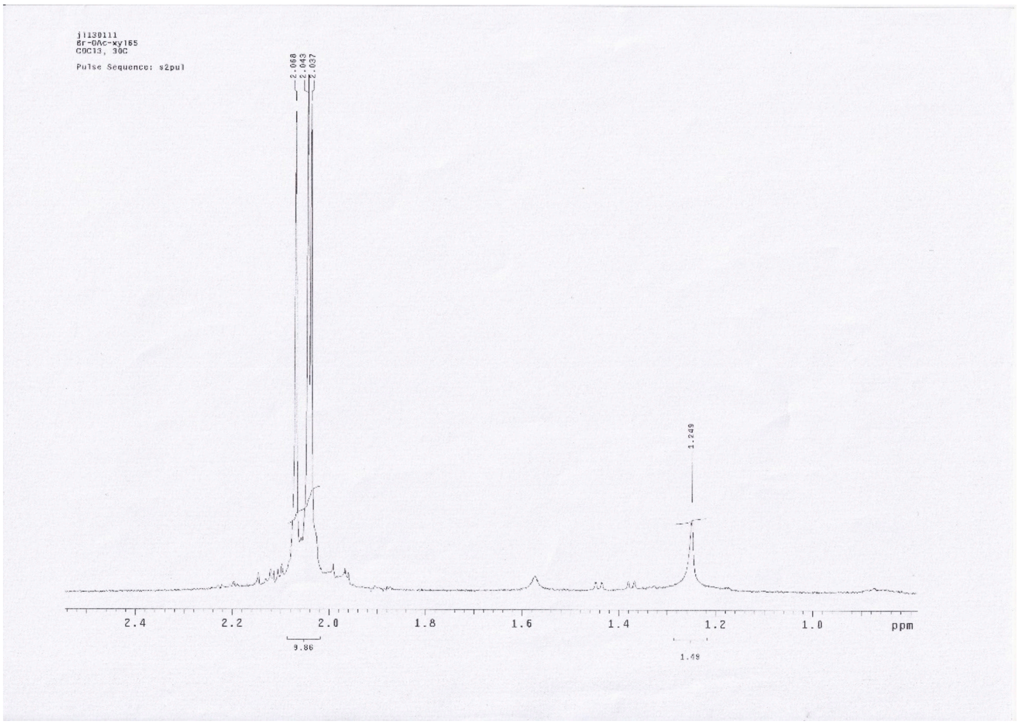

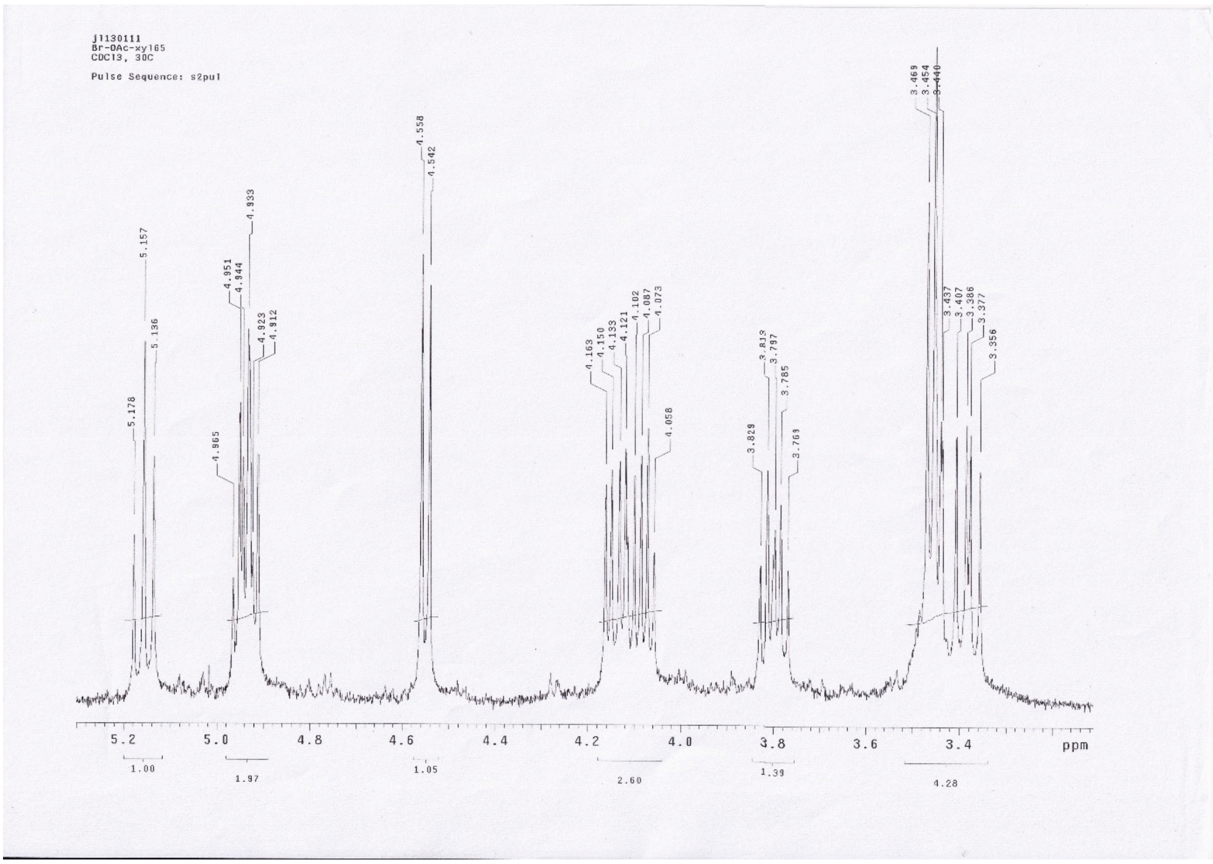
j1130111
Br-OAc-xyl65
CDCl3, 30C
Pulse Sequence: s2pul
5.178
5.157
5.136
4.965
4.951
4.944
4.933
4.923
4.912
4.558
4.542
4.163
4.150
4.133
4.121
4.102
4.087
4.073
4.058
3.829
3.813
3.797
3.785
3.769
3.469
3.454
3.440
3.437
3.407
3.386
3.377
3.356
5.2
5.0
4.8
4.6
4.4
4.2
4.0
3.8
3.6
3.4
ppm
1.00
1.97
1.05
2.60
1.39
4.28

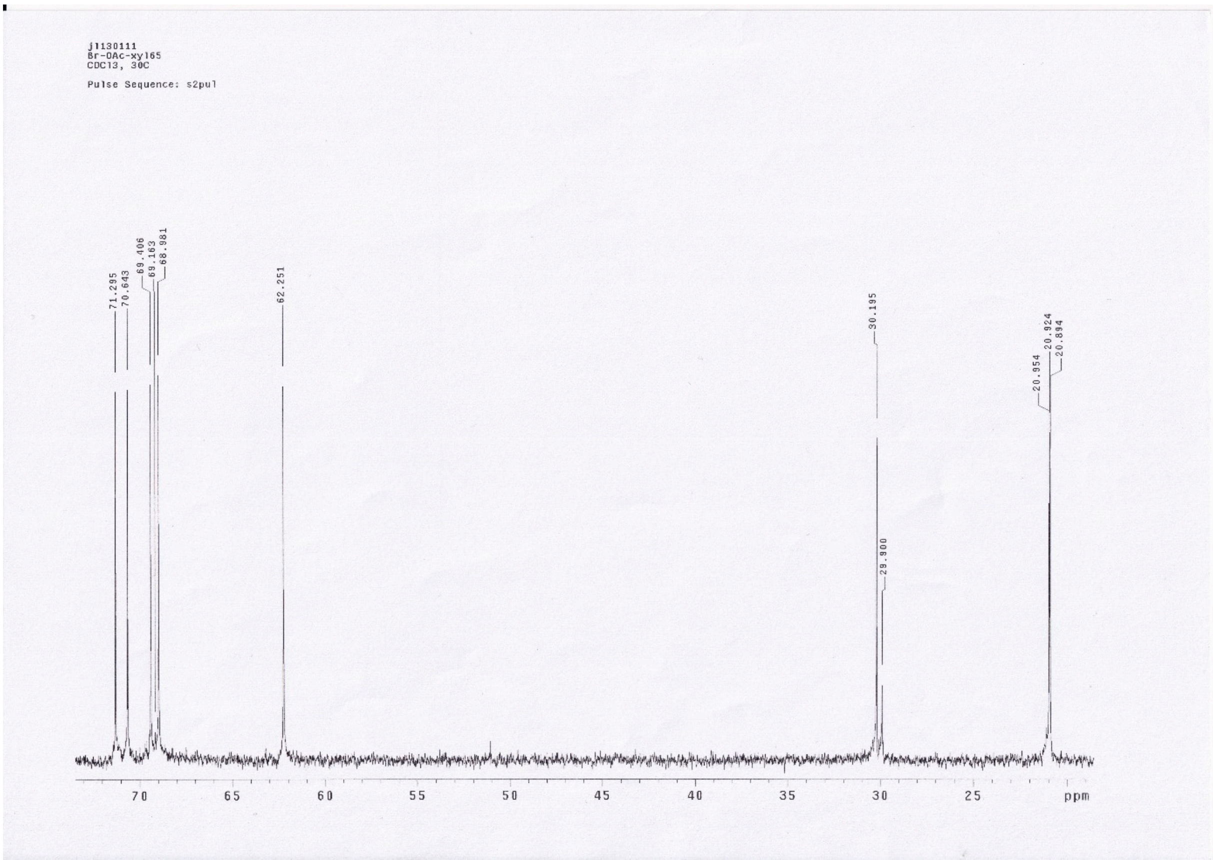
j1130111
Br-OAc-xyl65
CDCl3, 30C
Pulse Sequence: s2pul
71.295
70.643
69.406
69.163
68.981
62.251
30.195
29.900
20.954
20.924
20.894
70
65
60
55
50
45
40
35
30
25
ppm

^{13}C NMR (100 MHz, $CDCl_3$):

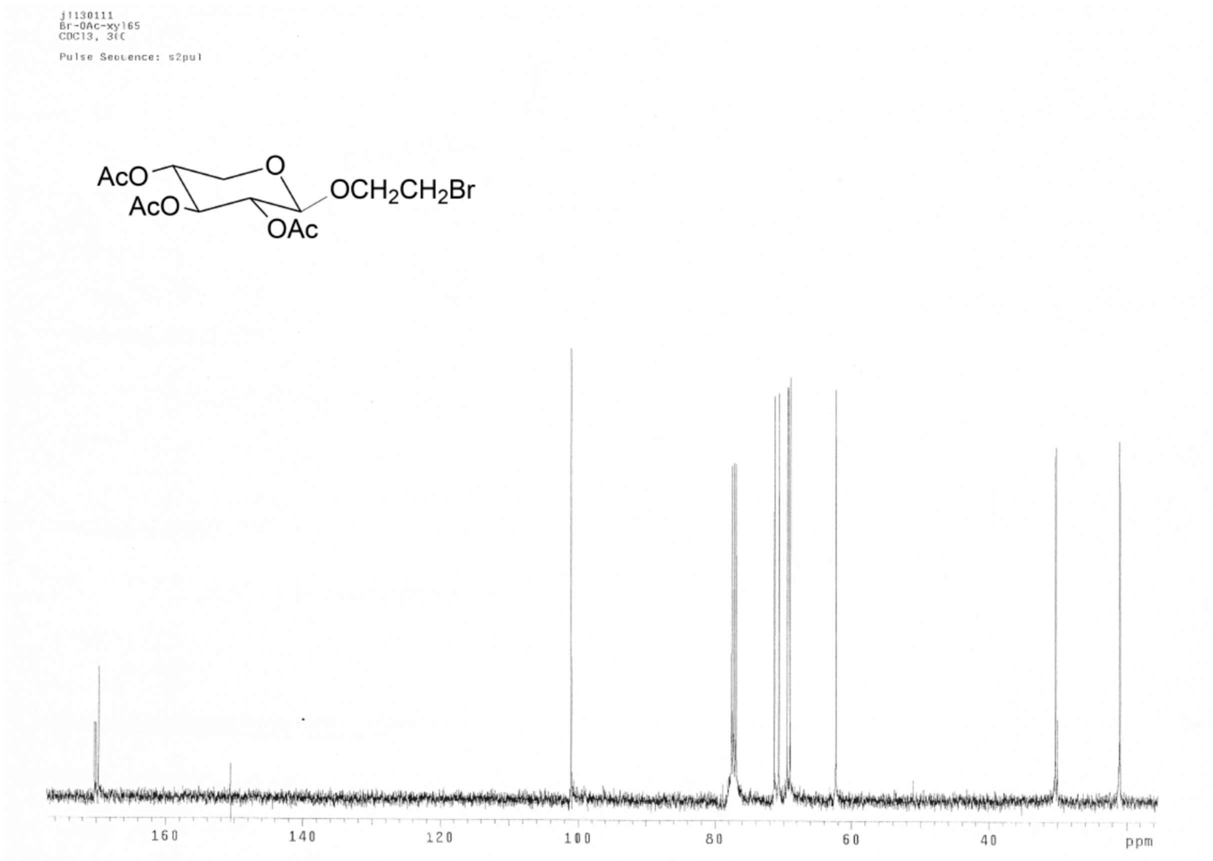

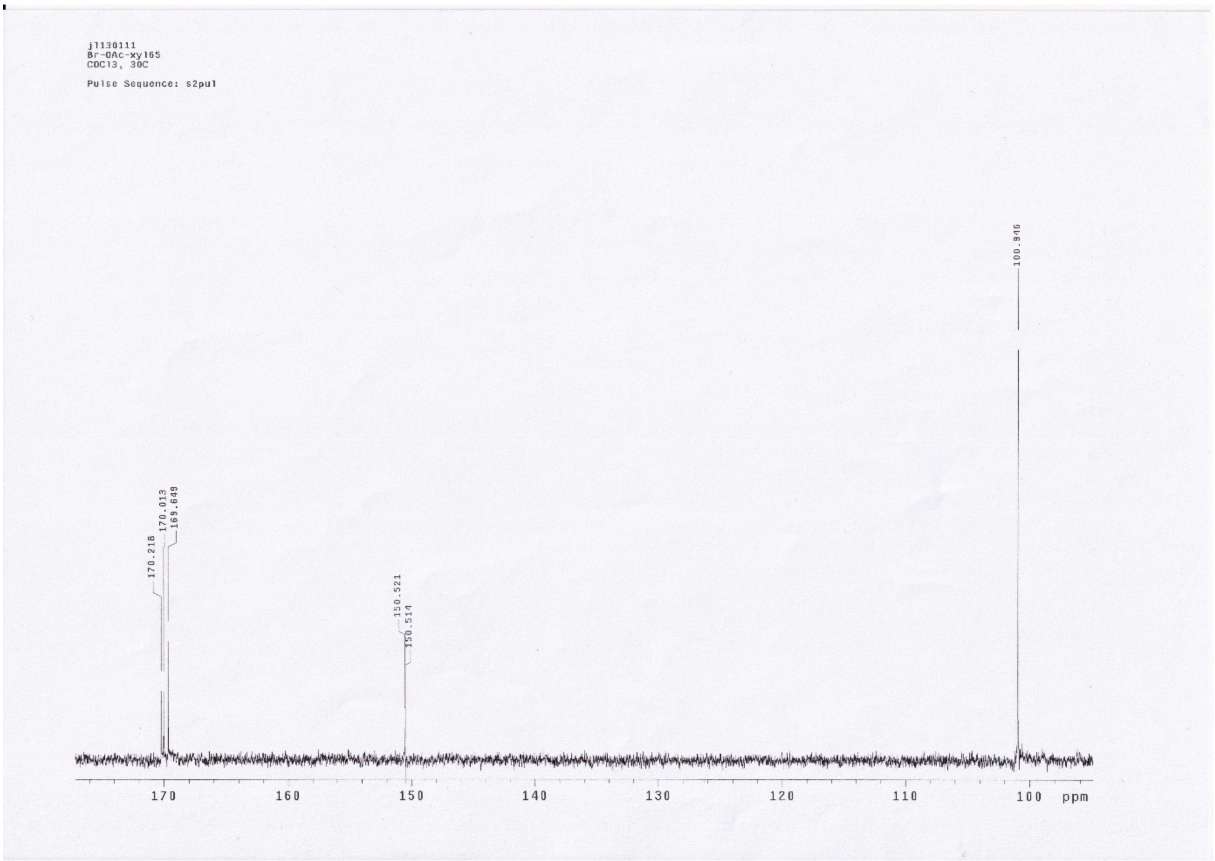

^{1}H-^{1}H COSY NMR:

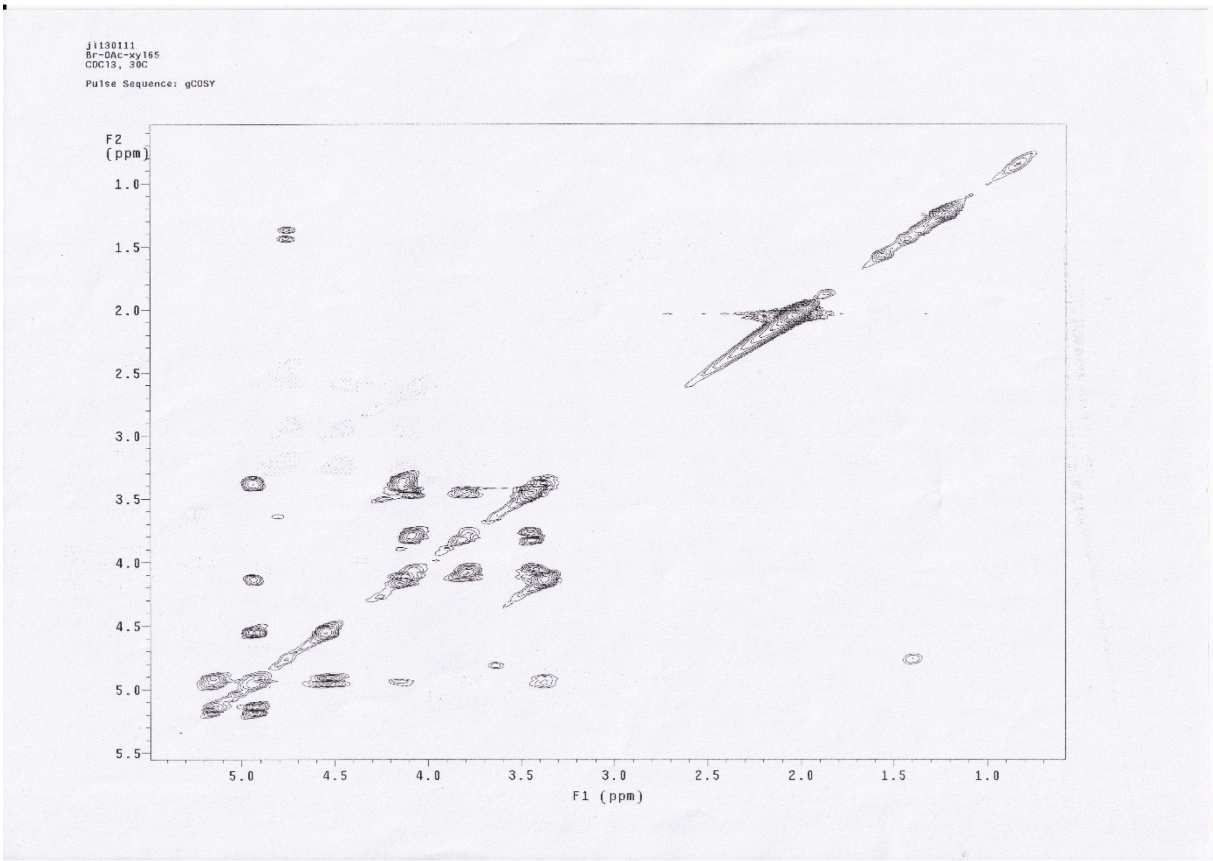

^{1}H-^{13}C HSQC NMR:

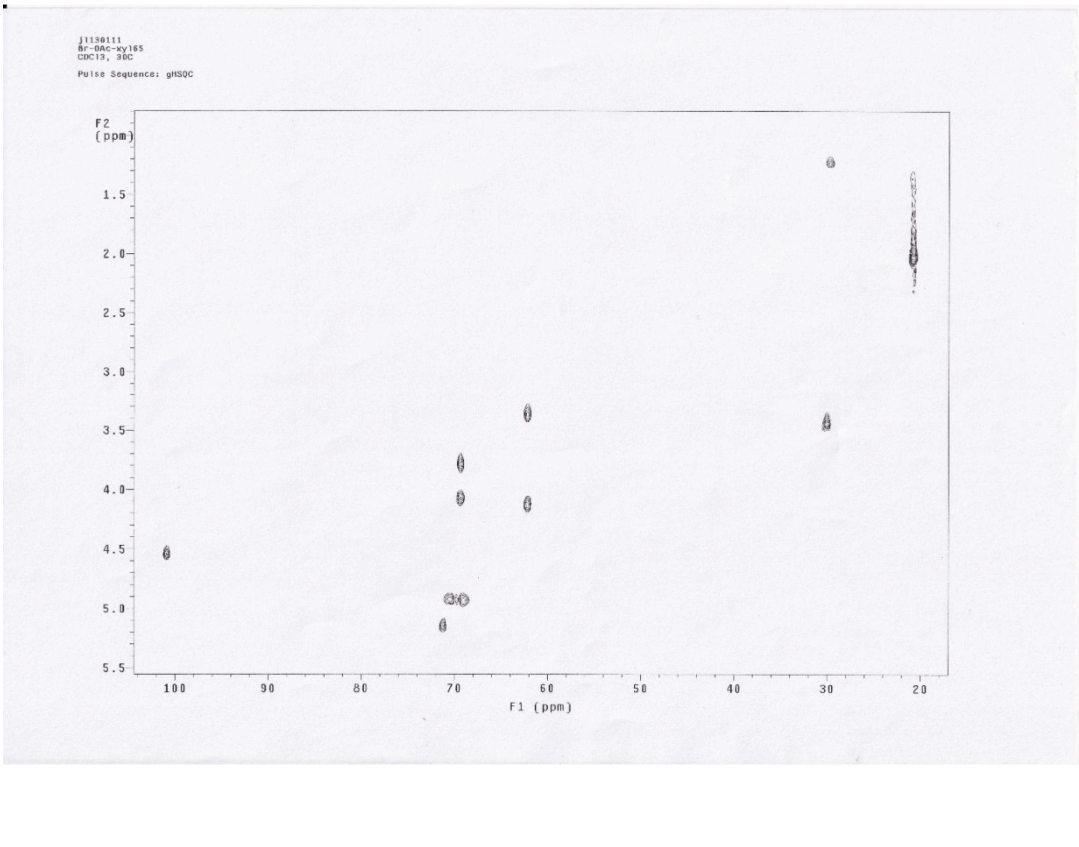

2-Bromoethyl 2′,3′,4′-tri-O-acetyl-α-D-xylopyranoside (**3**)

^{1}H NMR (400 MHz, $CDCl_3$):

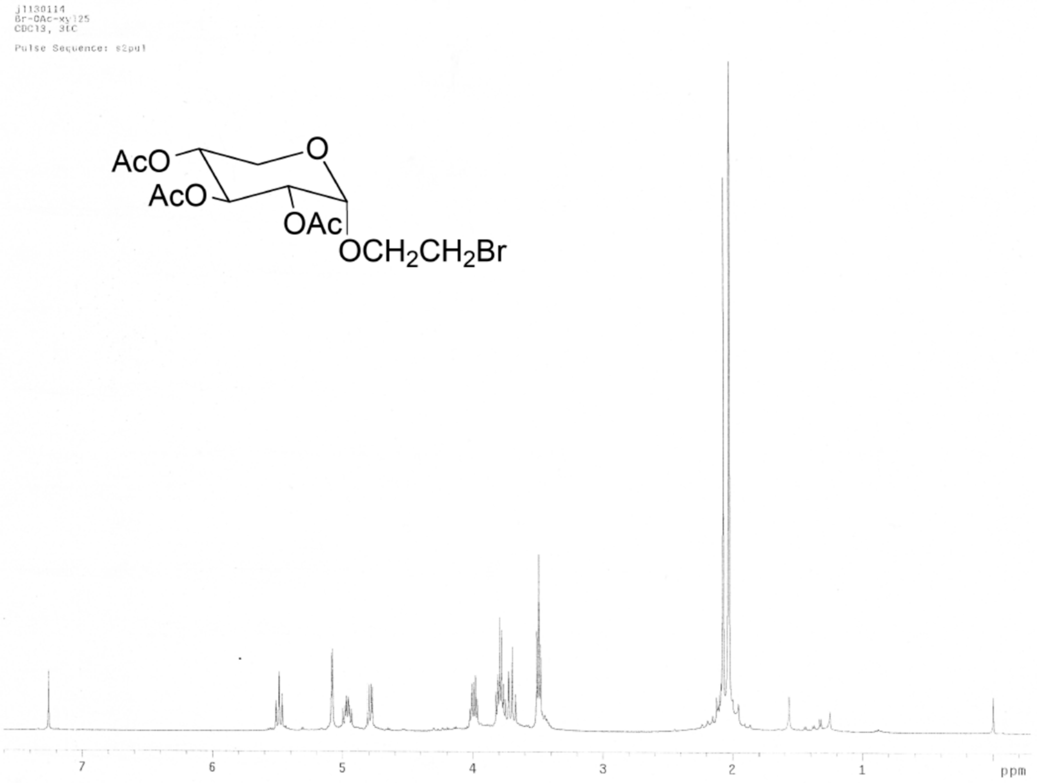

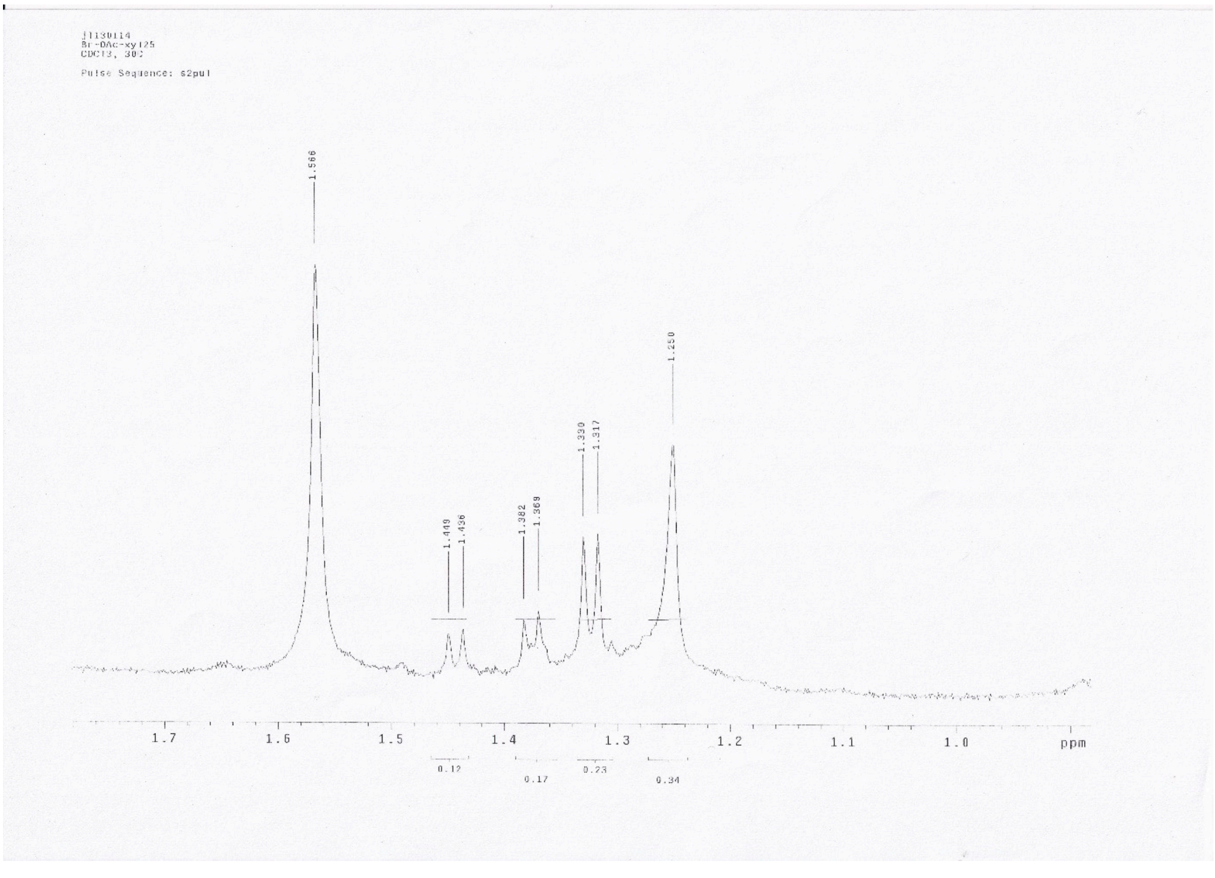

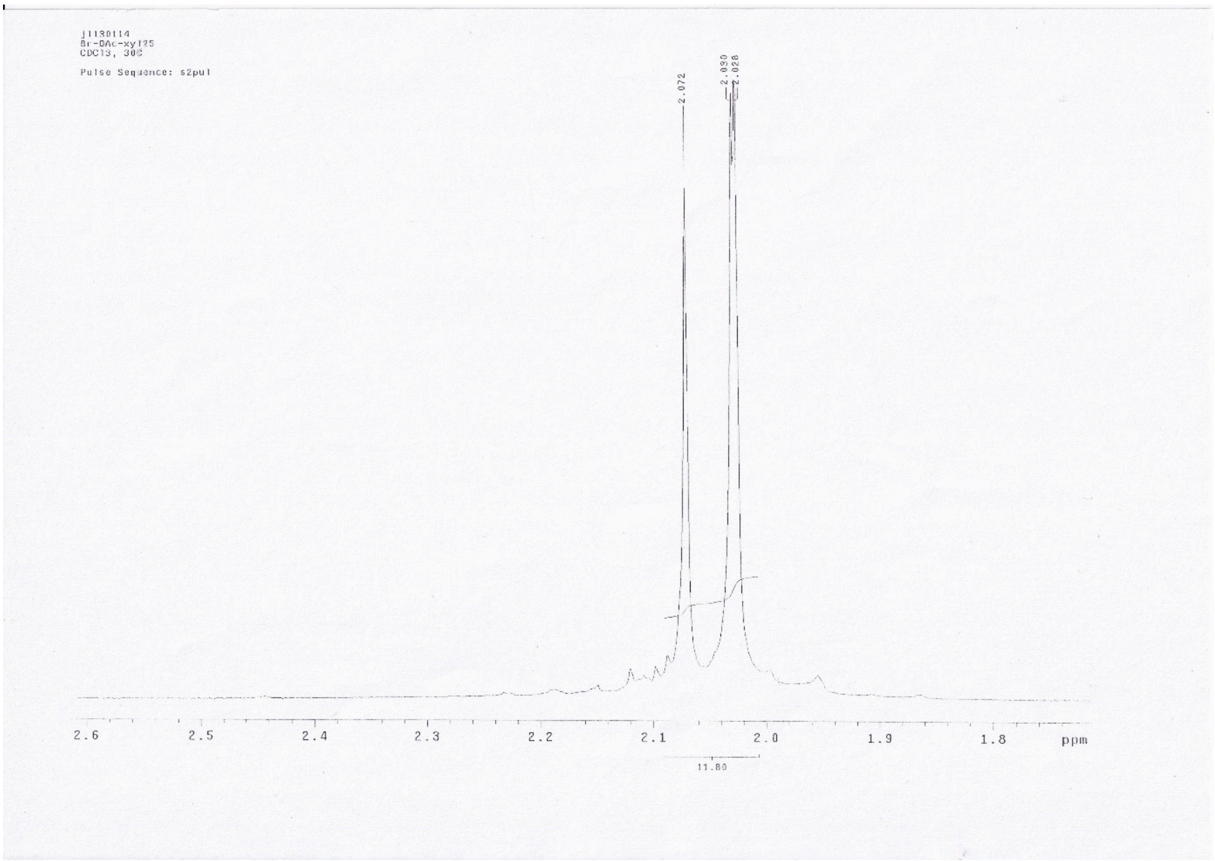

jl130114
Br-OAc-xyl25
CDCl3, 30C
Pulse Sequence: s2pul
2.072
2.030
2.028
2.6 2.5 2.4 2.3 2.2 2.1 2.0 1.9 1.8 ppm
11.80

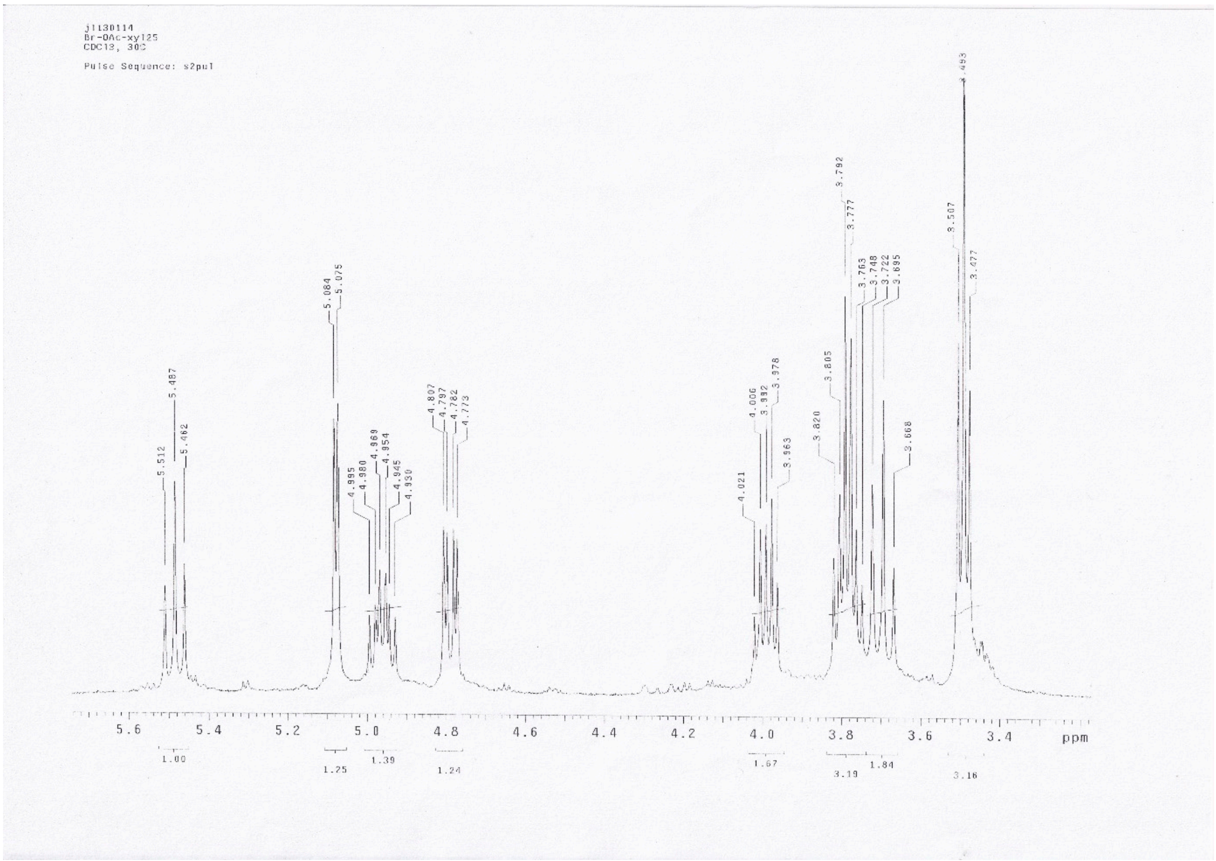

jl130114
Br-OAc-xyl25
CDCl3, 30C
Pulse Sequence: s2pul
5.512
5.487
5.462
5.084
5.075
4.995
4.980
4.969
4.954
4.945
4.930
4.807
4.797
4.782
4.773
4.021
4.006
3.992
3.978
3.963
3.820
3.805
3.792
3.777
3.763
3.748
3.722
3.695
3.668
3.507
3.493
3.477
5.6 5.4 5.2 5.0 4.8 4.6 4.4 4.2 4.0 3.8 3.6 3.4 ppm
1.00
1.25
1.39
1.24
1.67
3.19
1.84
3.16

^{13}C NMR (100 MHz, $CDCl_3$):

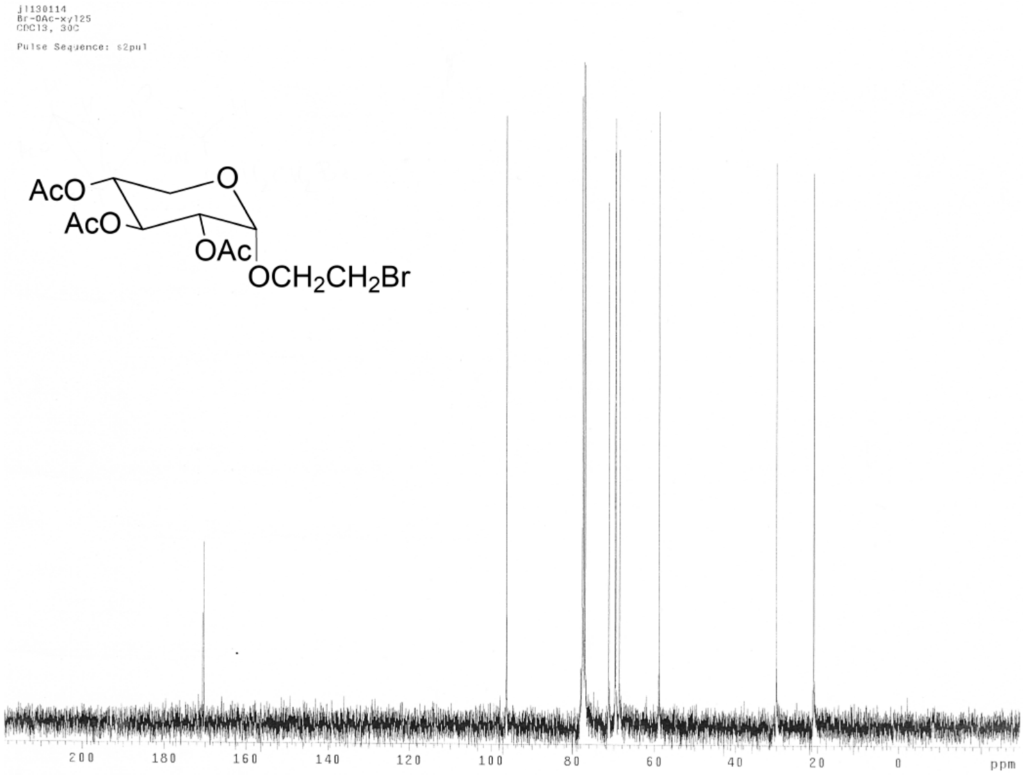

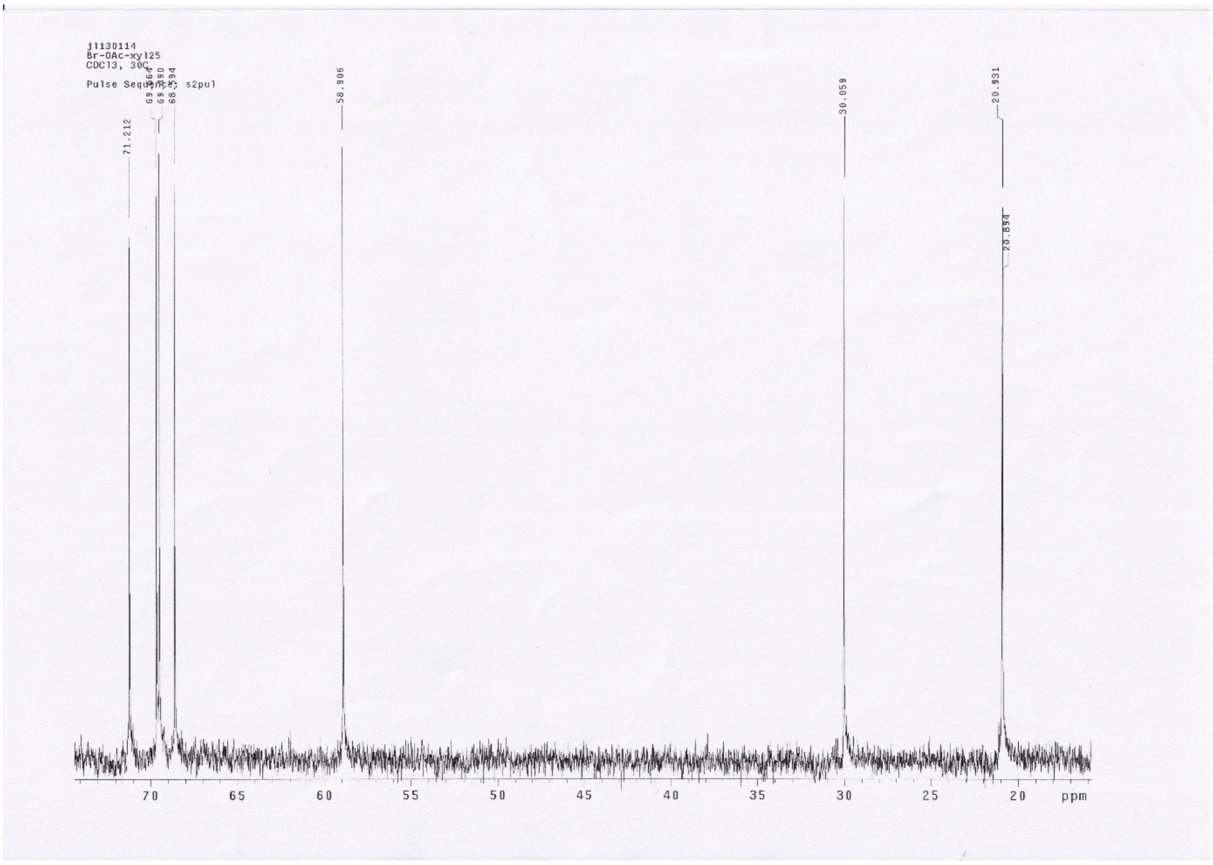

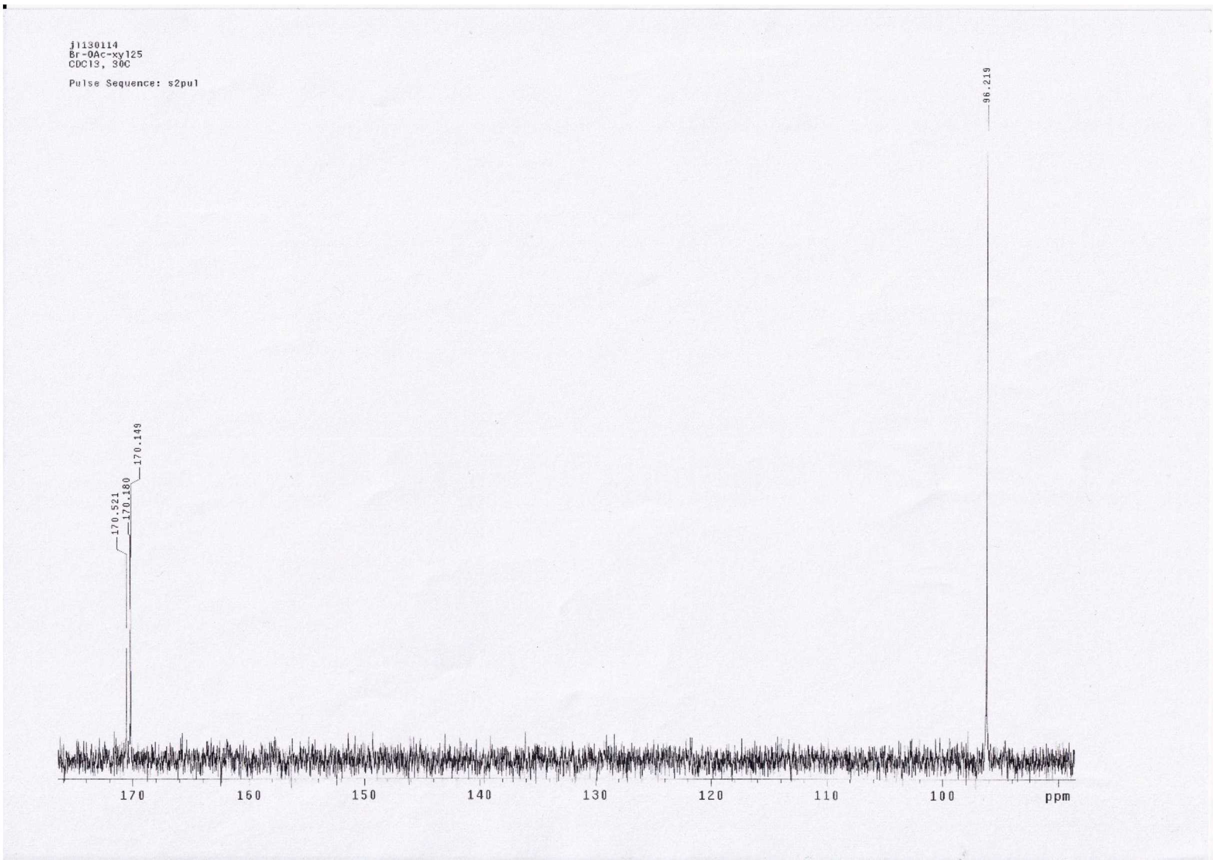

^1H-^1H COSY NMR:

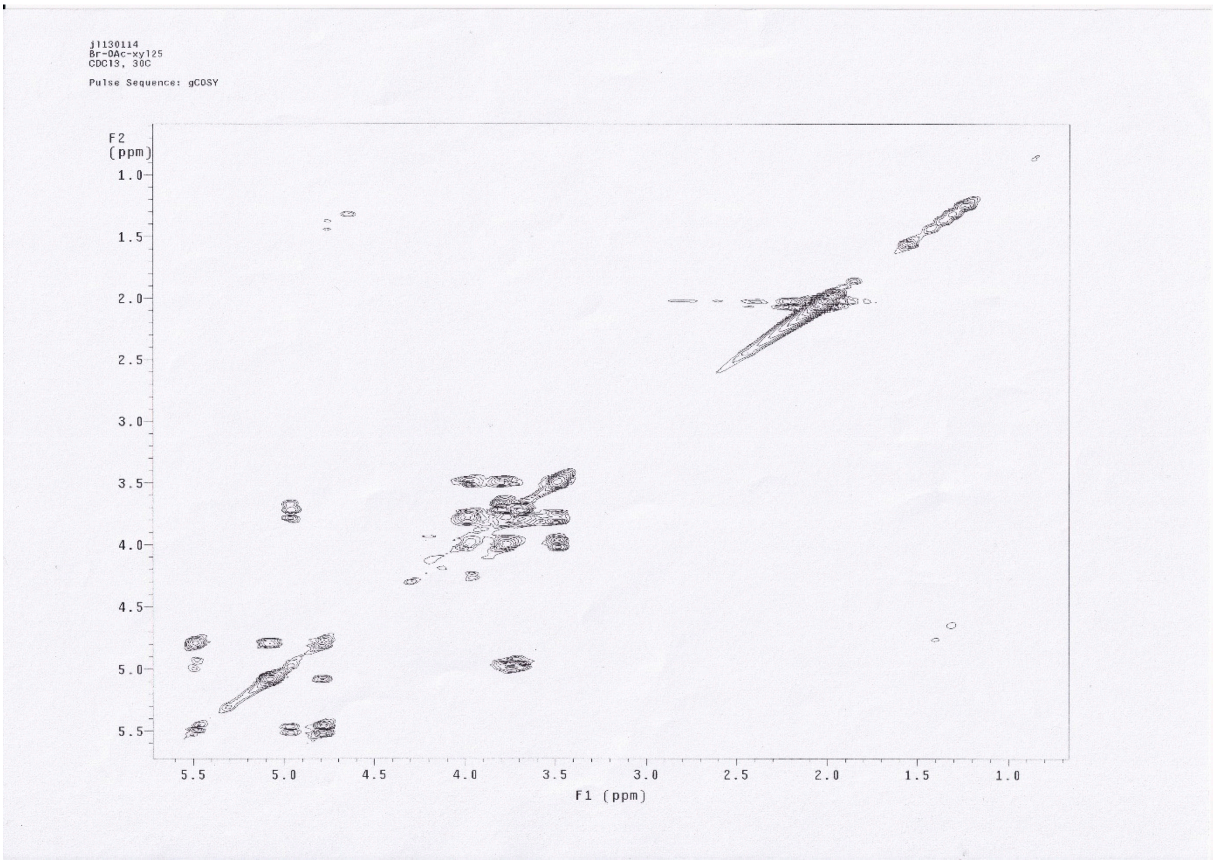

^{1}H-^{13}C HSQC NMR:

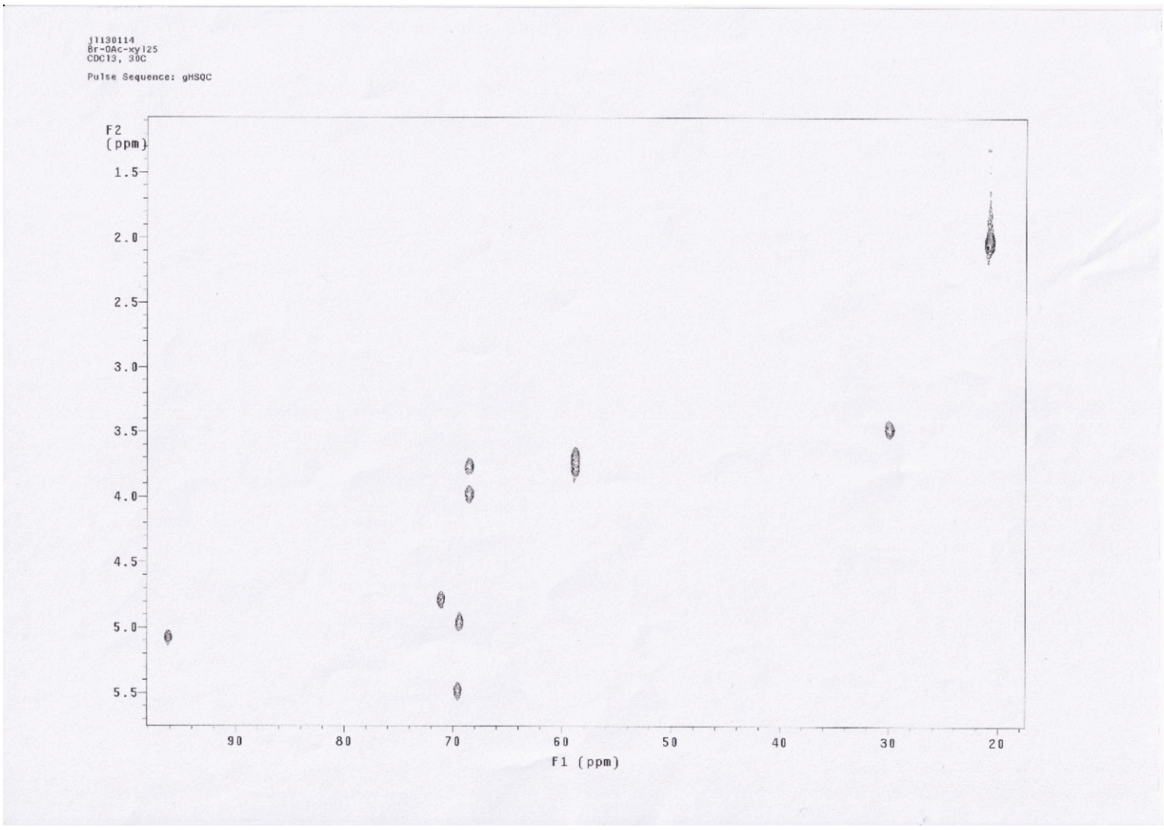

N-[2-(2′,3′,4′-tri-O-acetyl-β-D-xylopyranosyloxy)ethyl]pyridinium bromide (**4a**)

^{1}H NMR (500 MHz, D_2O):

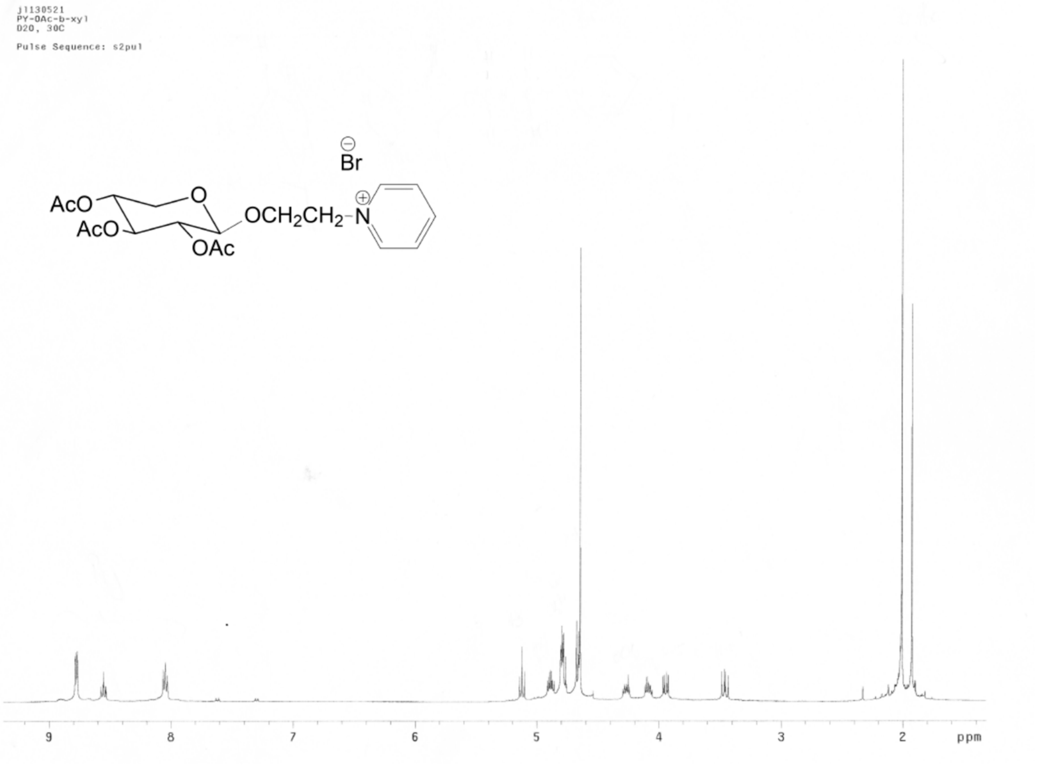

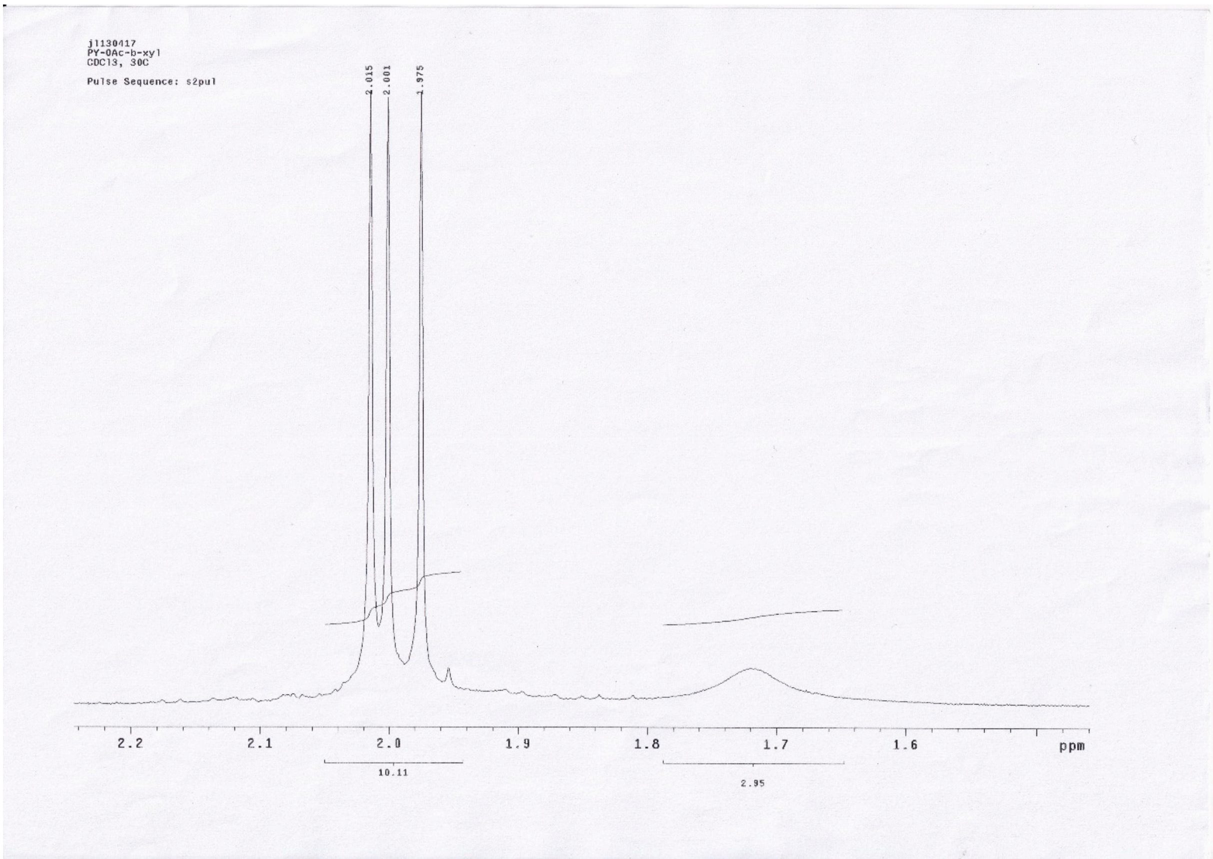
j1130417
PY-OAc-b-xyl
CDCl3, 30C
Pulse Sequence: s2pul
2.015
2.001
1.975
2.2
2.1
2.0
1.9
1.8
1.7
1.6
ppm
10.11
2.95

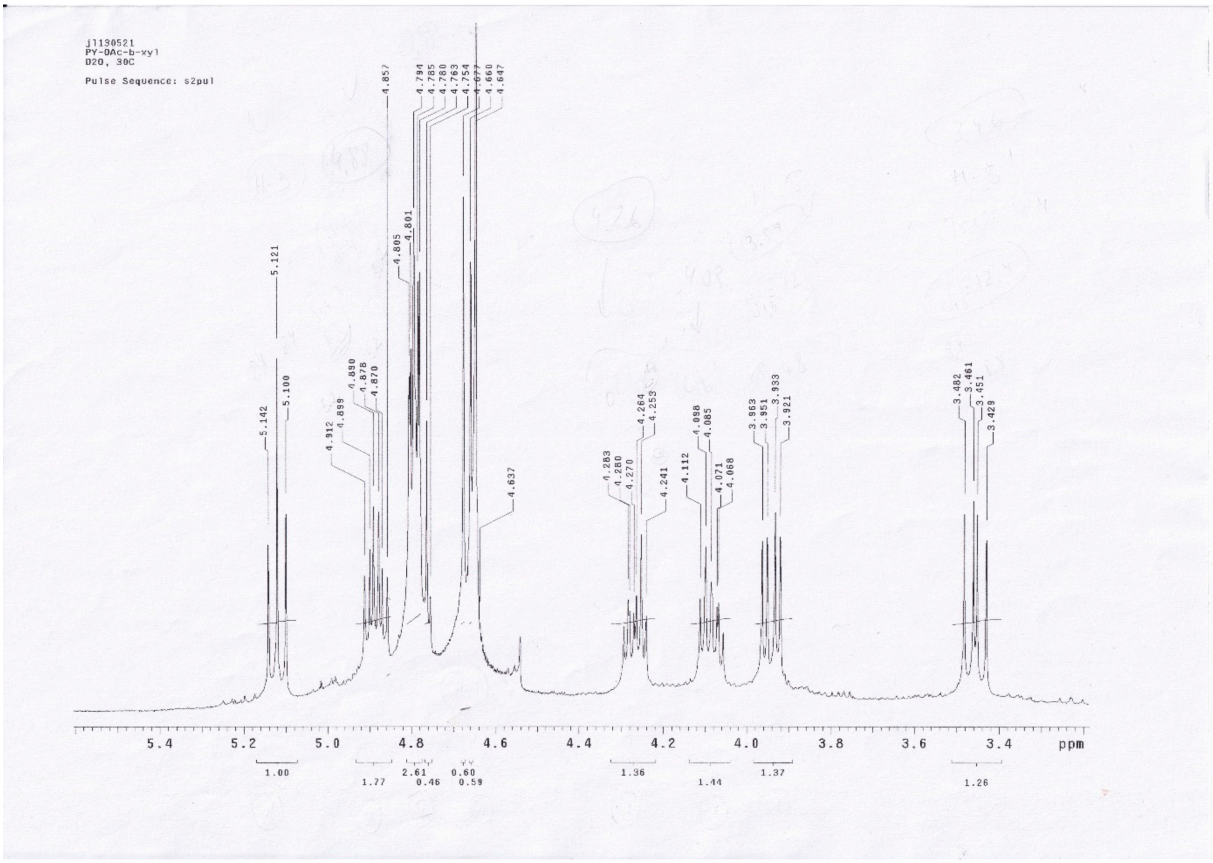
j1130521
PY-OAc-b-xyl
D2O, 30C
Pulse Sequence: s2pul
5.142
5.121
5.100
4.912
4.899
4.890
4.878
4.870
4.857
4.805
4.801
4.794
4.785
4.780
4.763
4.754
4.677
4.660
4.647
4.637
4.283
4.280
4.270
4.264
4.253
4.241
4.112
4.098
4.085
4.071
4.068
3.963
3.951
3.933
3.921
3.482
3.461
3.451
3.429
5.4
5.2
5.0
4.8
4.6
4.4
4.2
4.0
3.8
3.6
3.4
ppm
1.00
1.77
2.61
0.46
0.60
0.59
1.36
1.44
1.37
1.26

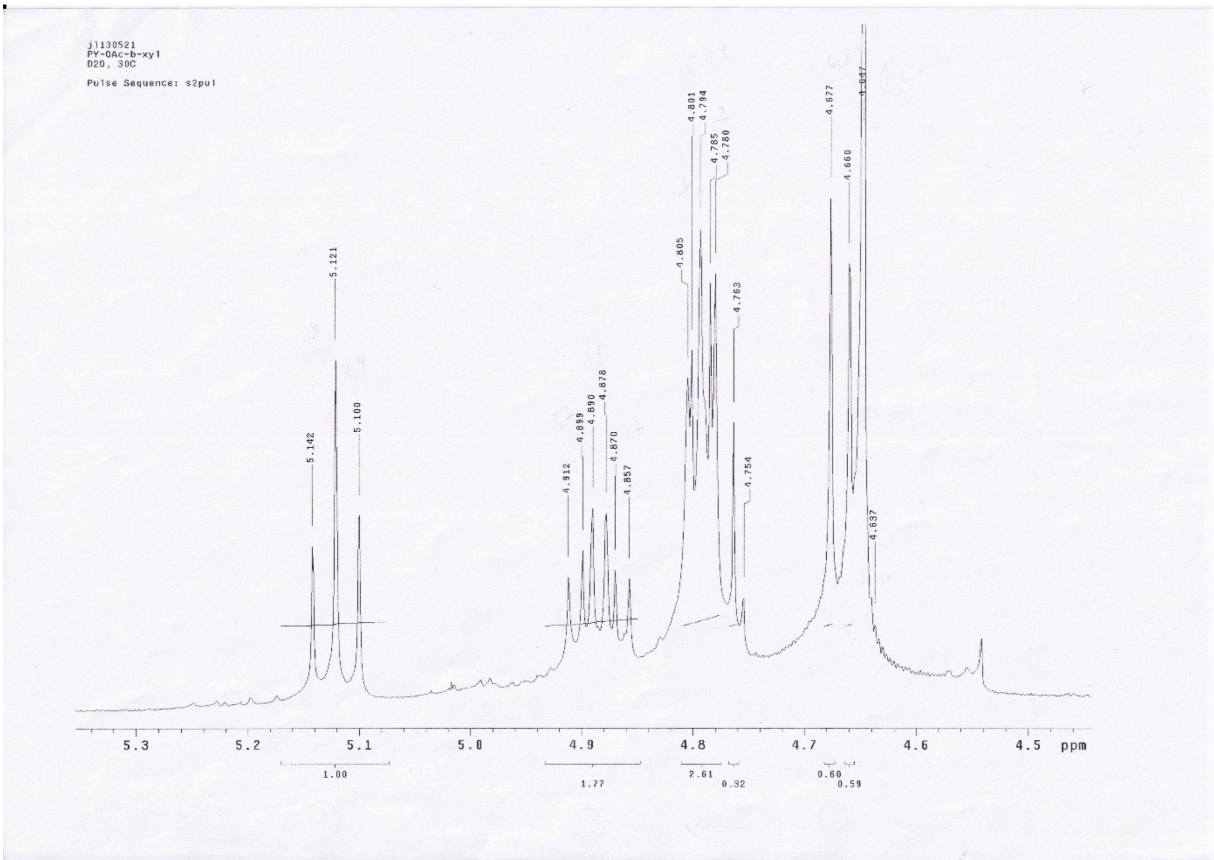

jl130521
PY-OAc-b-xyl
D2O, 30C
Pulse Sequence: s2pul
5.142
5.121
5.100
4.912
4.899
4.890
4.878
4.870
4.857
4.805
4.801
4.794
4.785
4.780
4.763
4.754
4.677
4.660
4.647
4.637
5.3
5.2
5.1
5.0
4.9
4.8
4.7
4.6
4.5
ppm
1.00
1.77
2.61
0.32
0.60
0.59

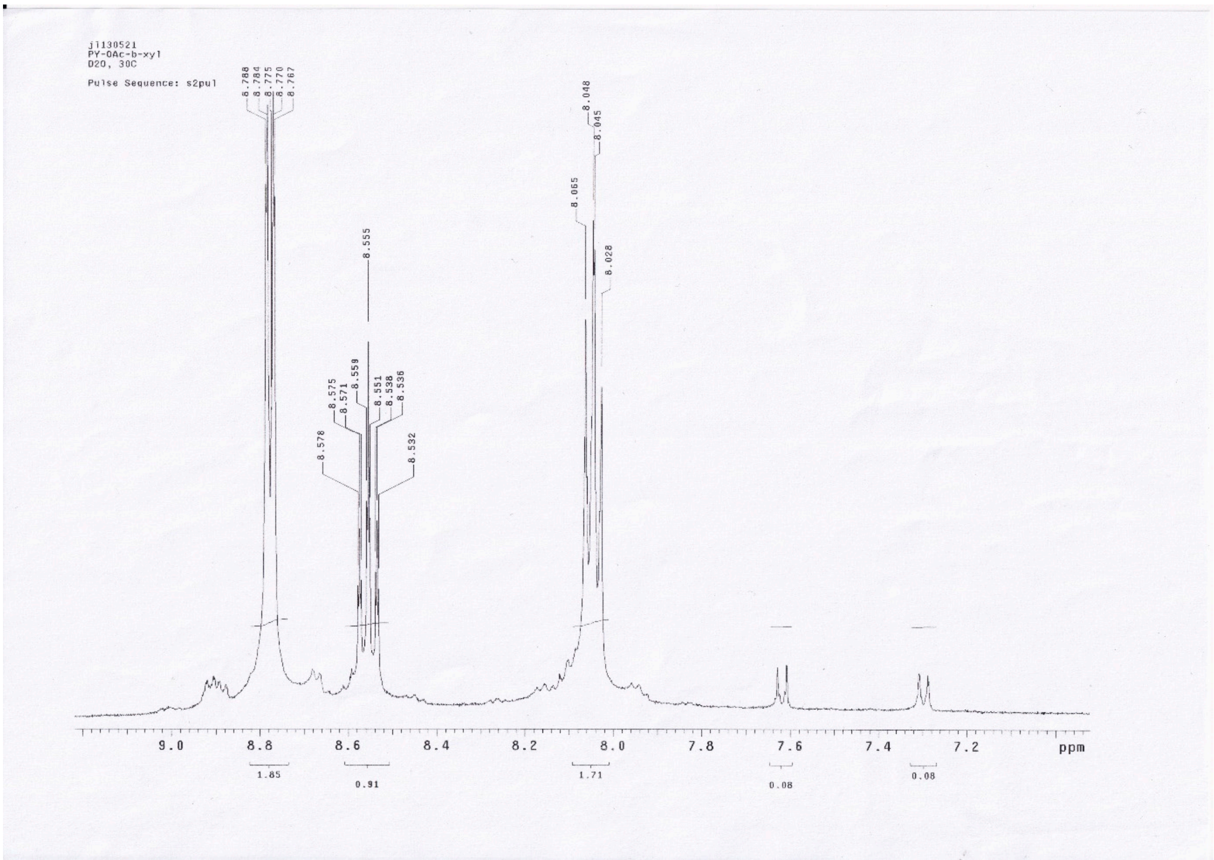

jl130521
PY-OAc-b-xyl
D2O, 30C
Pulse Sequence: s2pul
8.788
8.784
8.775
8.770
8.767
8.578
8.575
8.571
8.559
8.555
8.551
8.538
8.536
8.532
8.065
8.048
8.045
8.028
9.0
8.8
8.6
8.4
8.2
8.0
7.8
7.6
7.4
7.2
ppm
1.85
0.91
1.71
0.08
0.08

^{13}C NMR (125 MHz, D_2O):

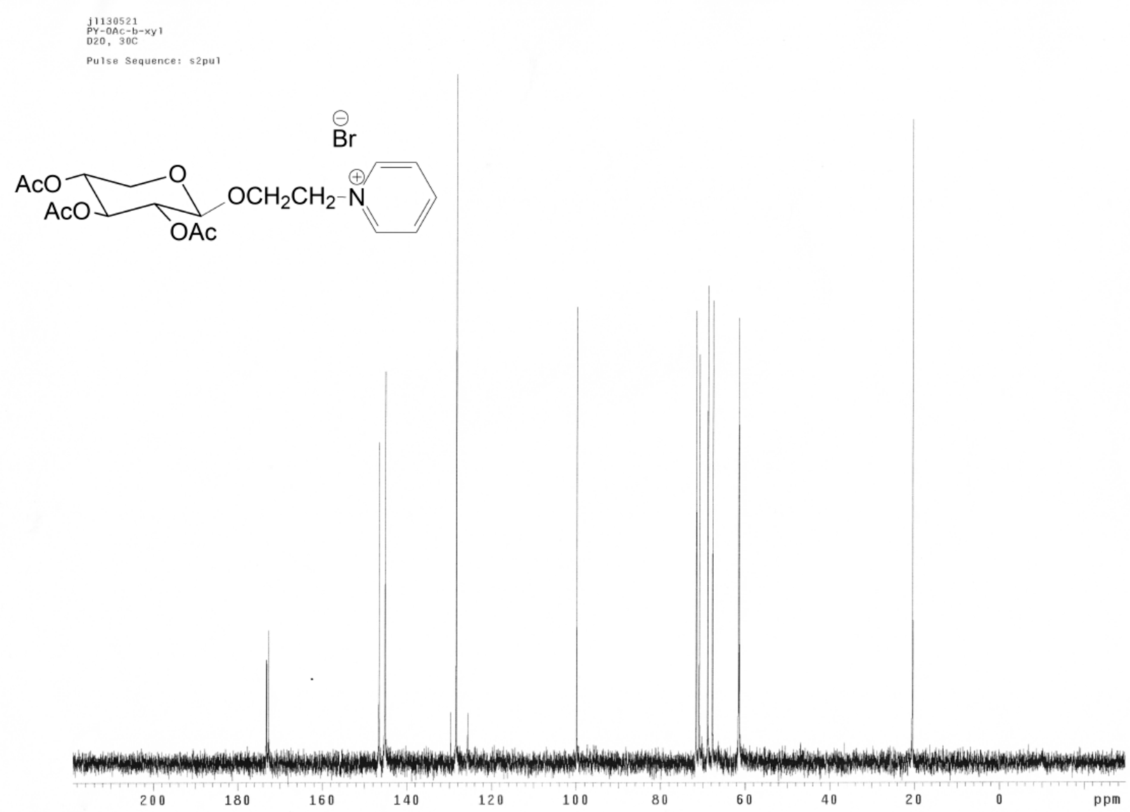

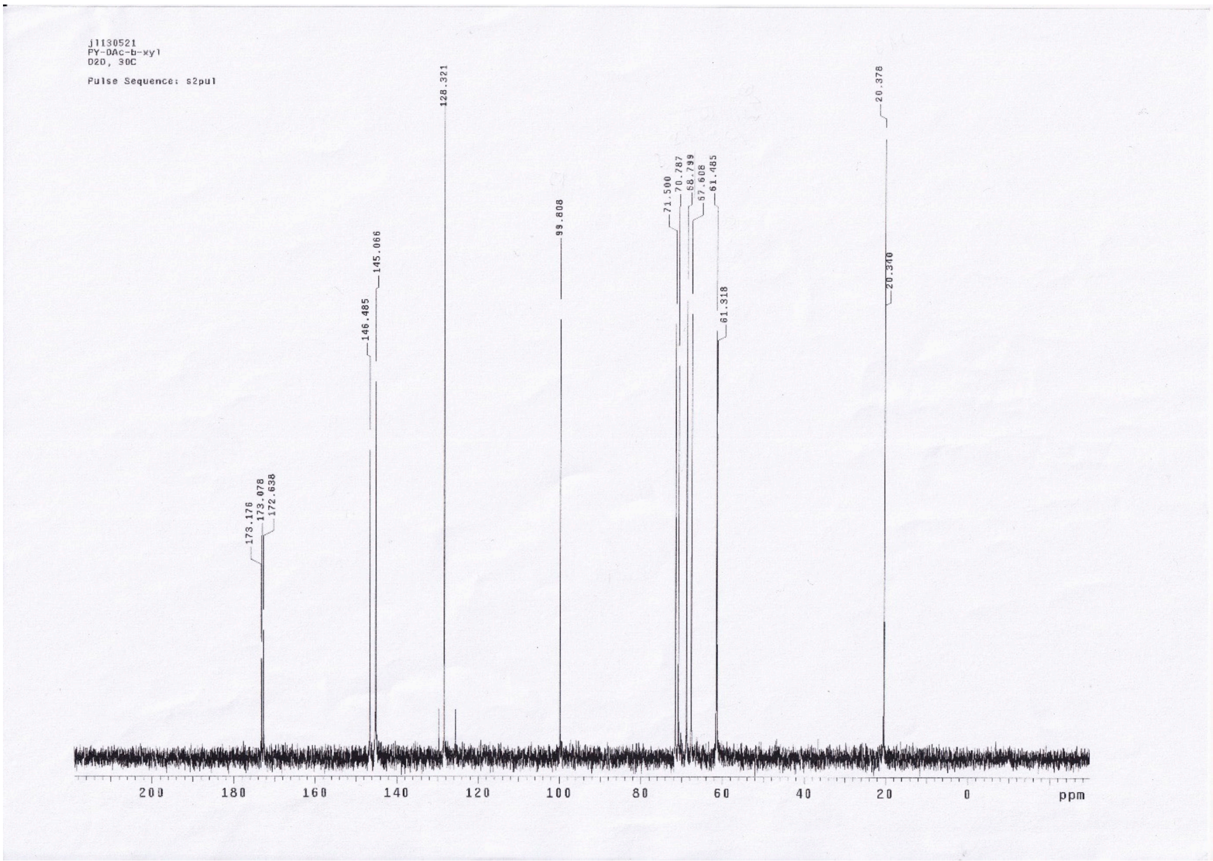

^{1}H-^{1}H COSY NMR:

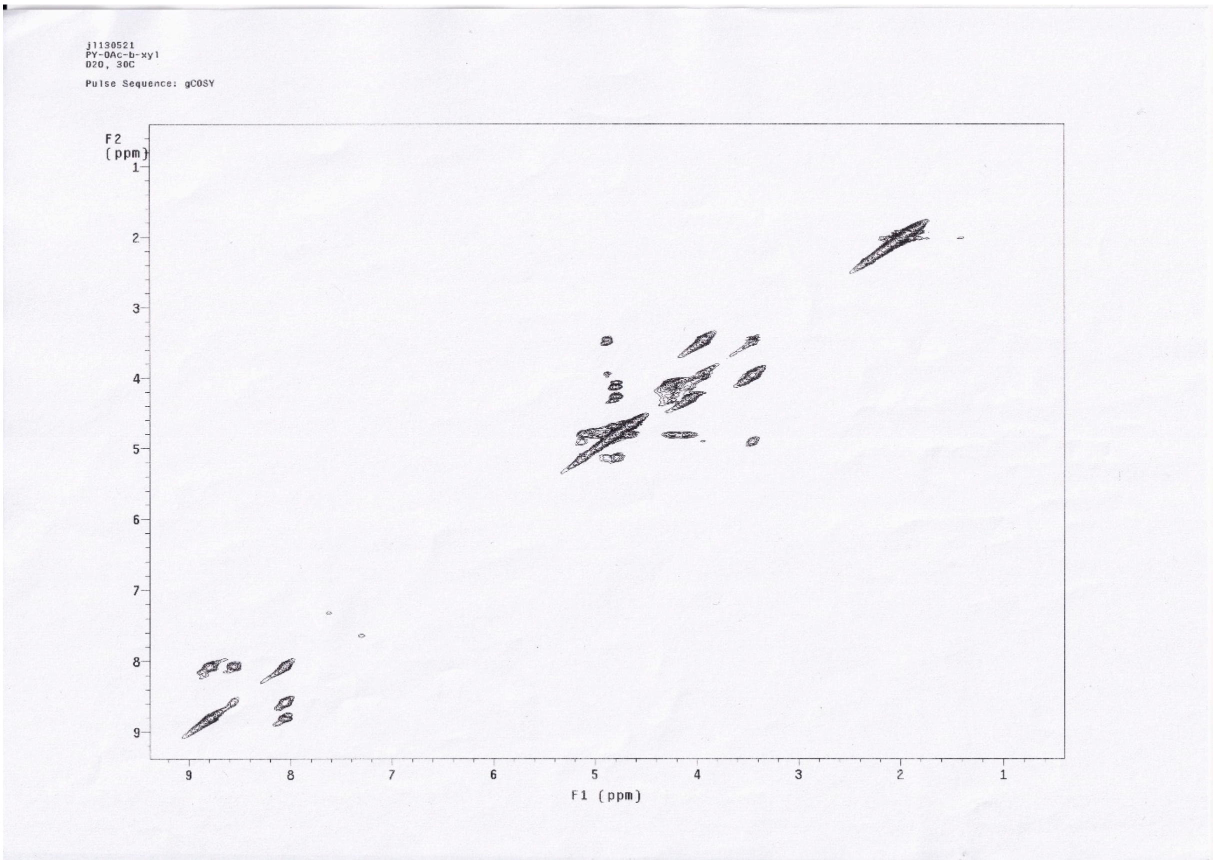

^{1}H-^{13}C HSQC NMR:

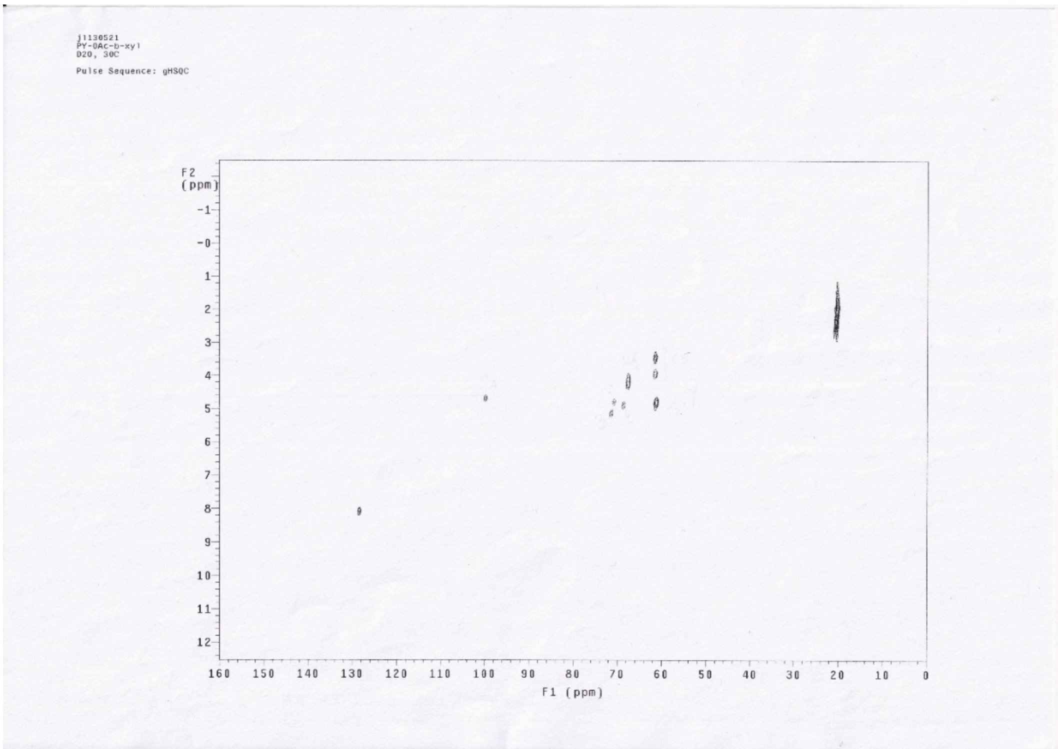

N-[2-(2′,3′,4′-tri-O-acetyl-β-D-xylopyranosyloxy)ethyl]]-*N*,*N*,*N*-trimethylammonium bromide (**4b**)

^{1}H NMR (500 MHz, D_2O):

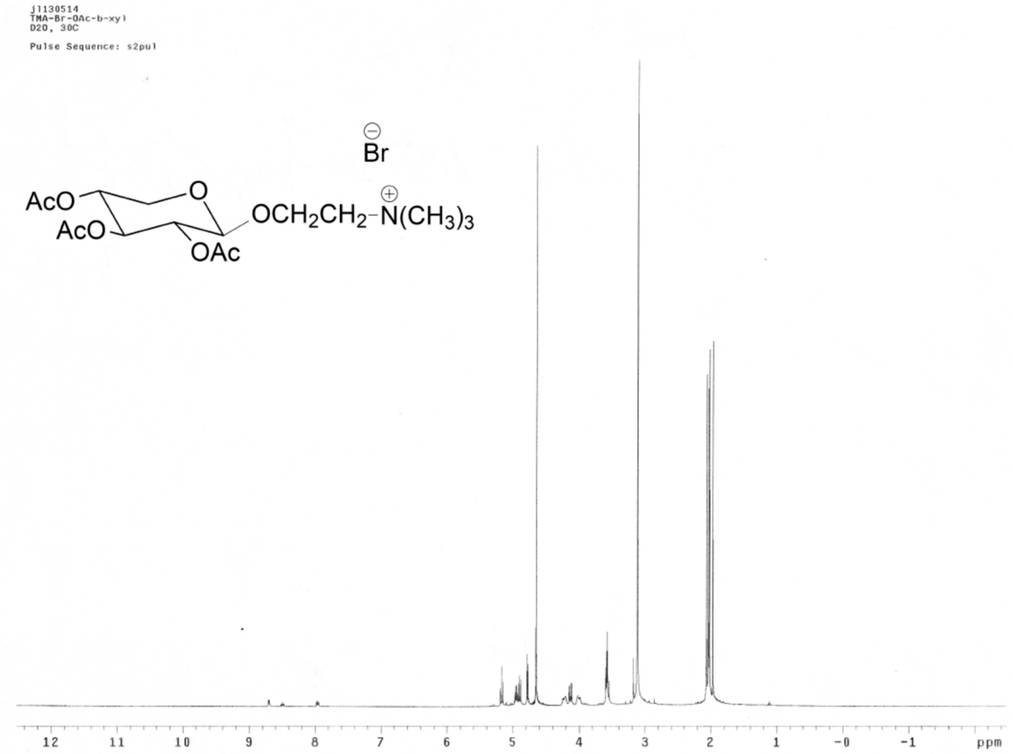

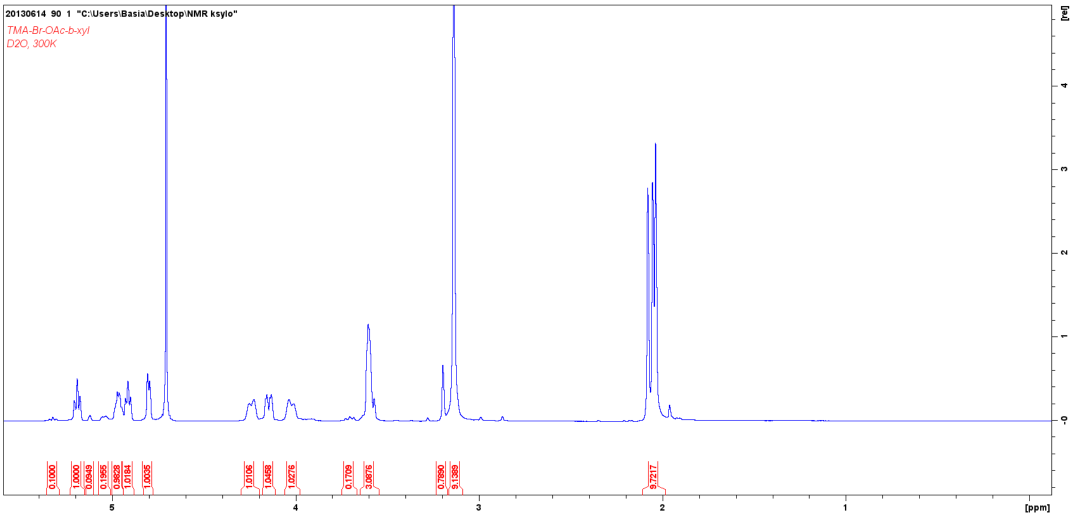

^{13}C NMR (125 MHz, D_2O):

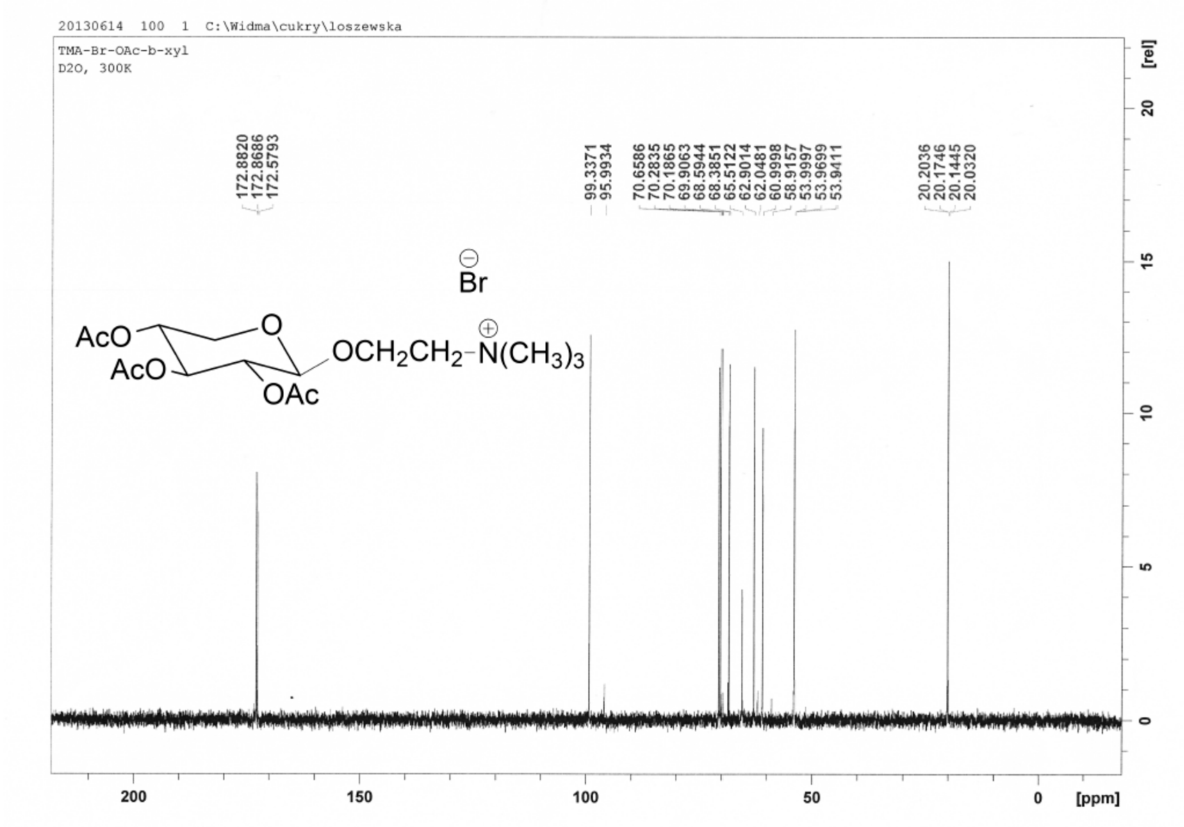

^{1}H-^{1}H COSY NMR:

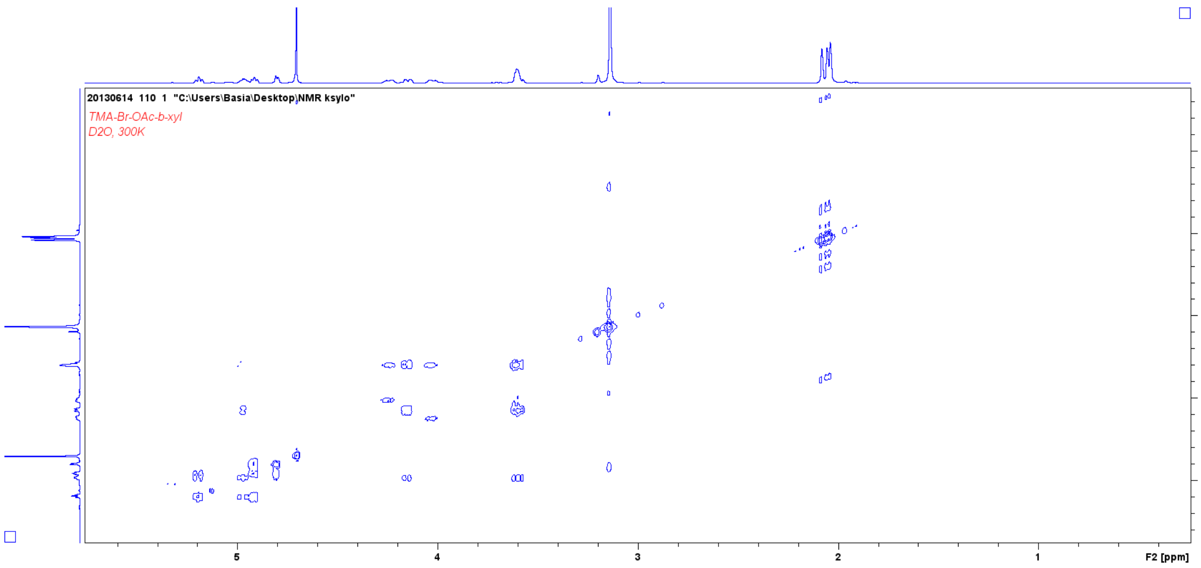

^{1}H-^{13}C HSQC NMR:

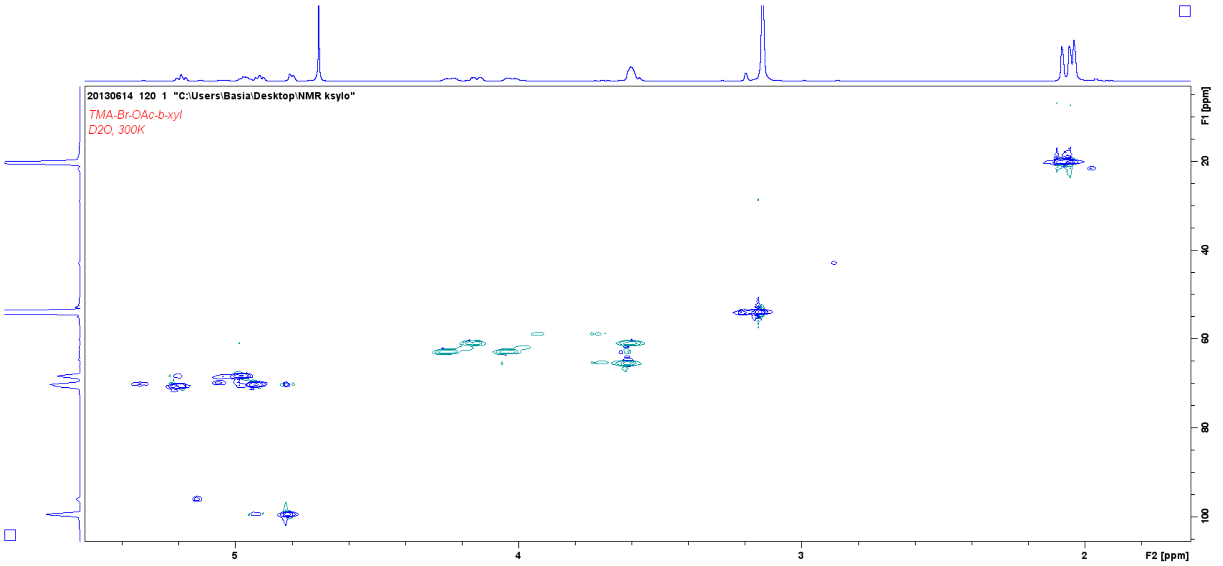

N-[2-(2′,3′,4′-tri-O-acetyl-β-D-xylopyranosyloxy)ethyl]-*N*-hexyl-*N*,*N*-dimethylammonium bromide (**4c**)

^{1}H NMR (500 MHz, D_2O):

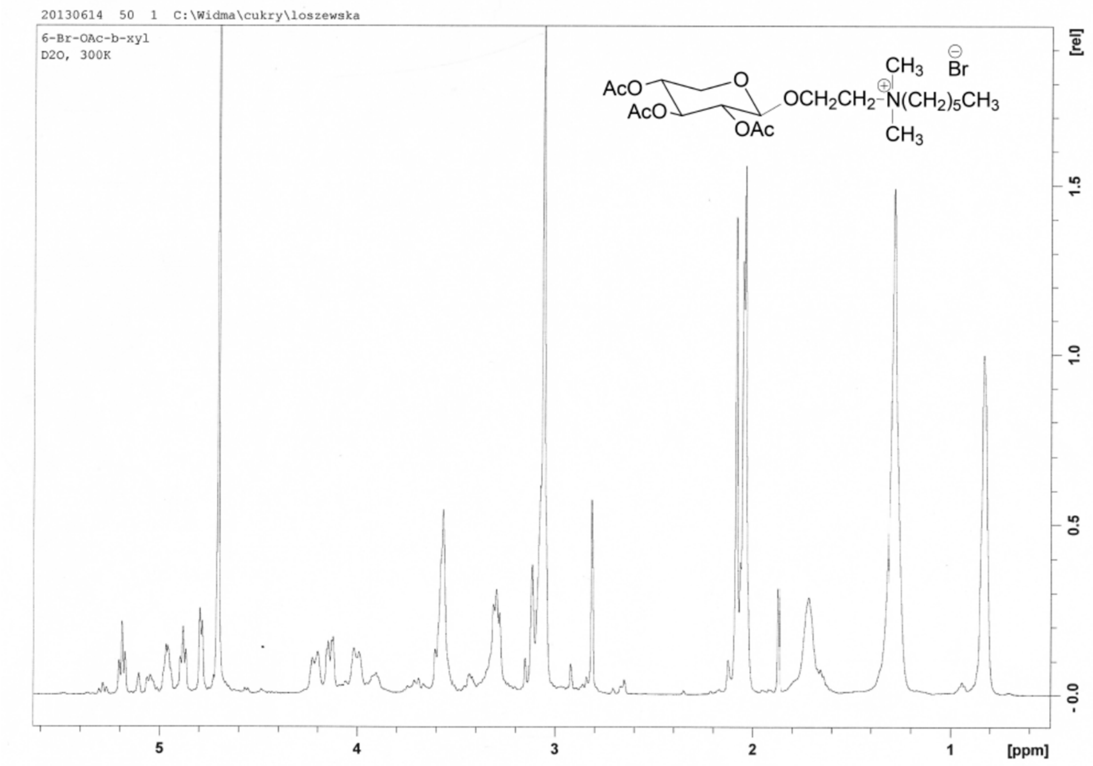

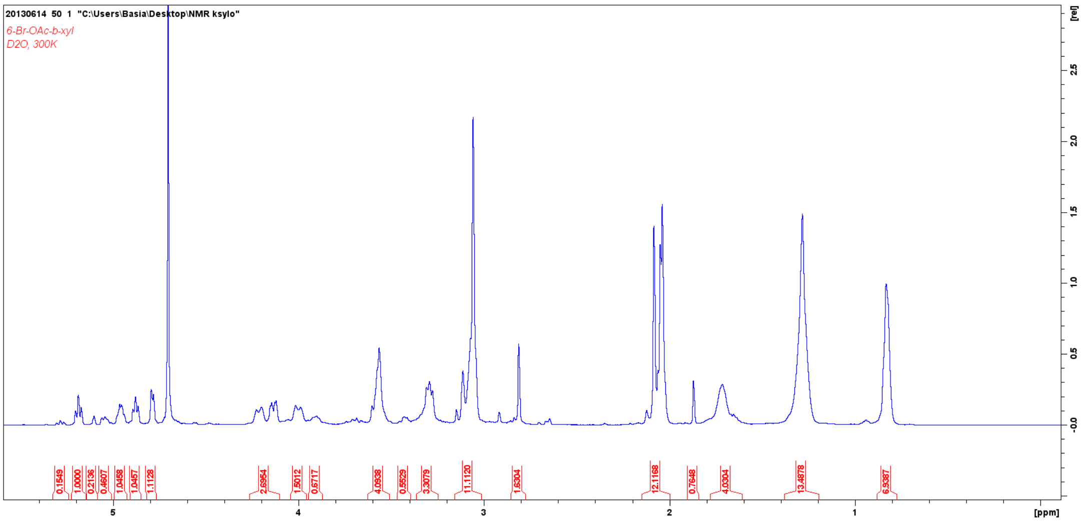

^{13}C NMR (125 MHz, D_2O):

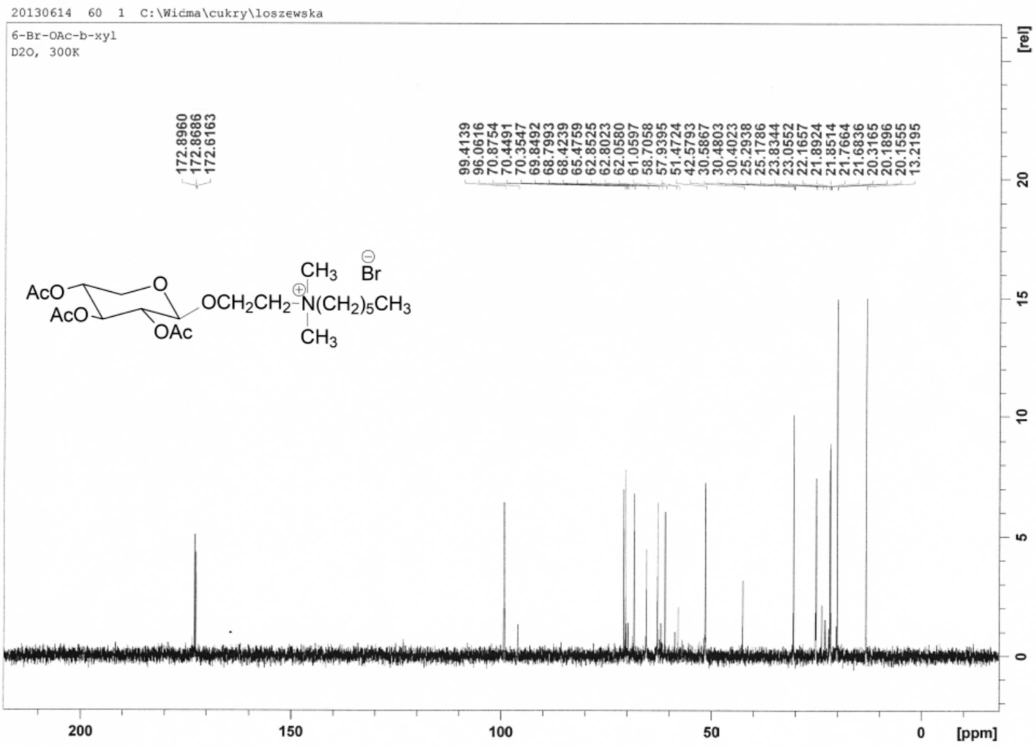

^{1}H-^{1}H COSY NMR:

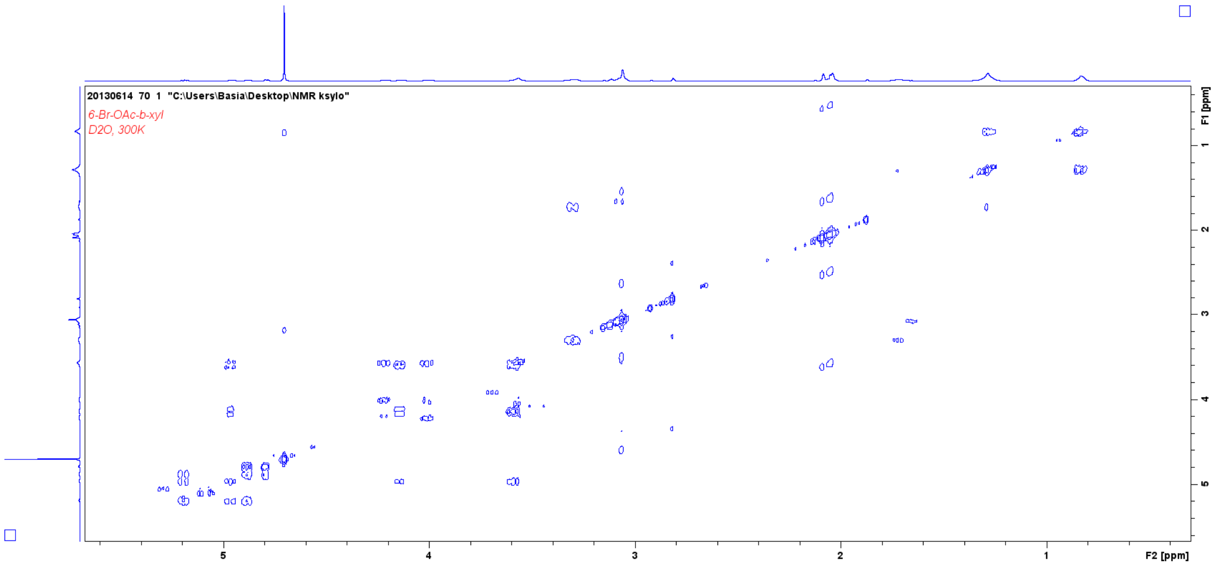

^{1}H-^{13}C HSQC NMR:

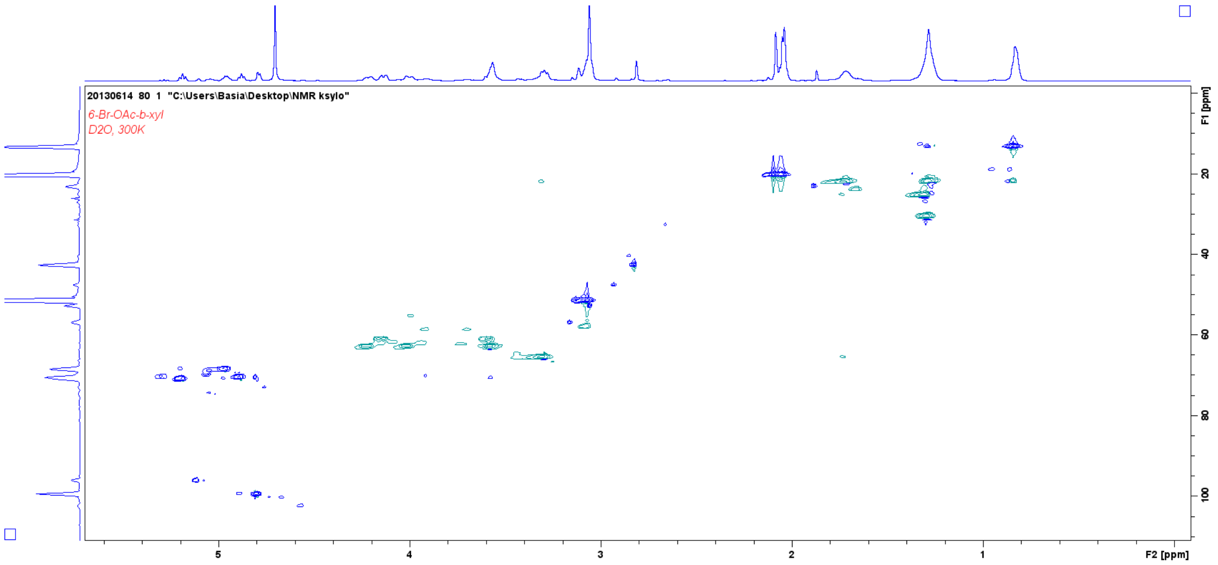

N-[2-(2′,3′,4′-tri-O-acetyl-β-D-xylopyranosyloxy)ethyl]-*N*,*N*-dimethyl-*N*-octylammonium bromide (**4d**)

^{1}H NMR (500 MHz, D_2O):

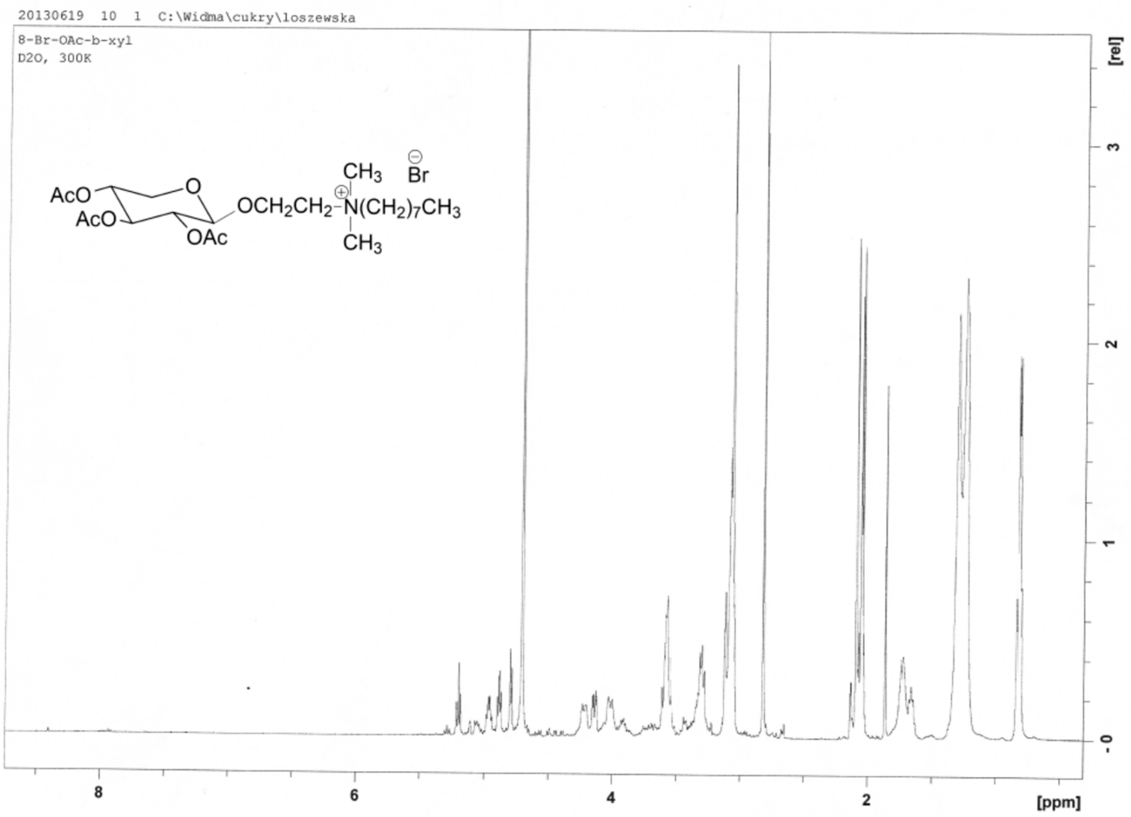

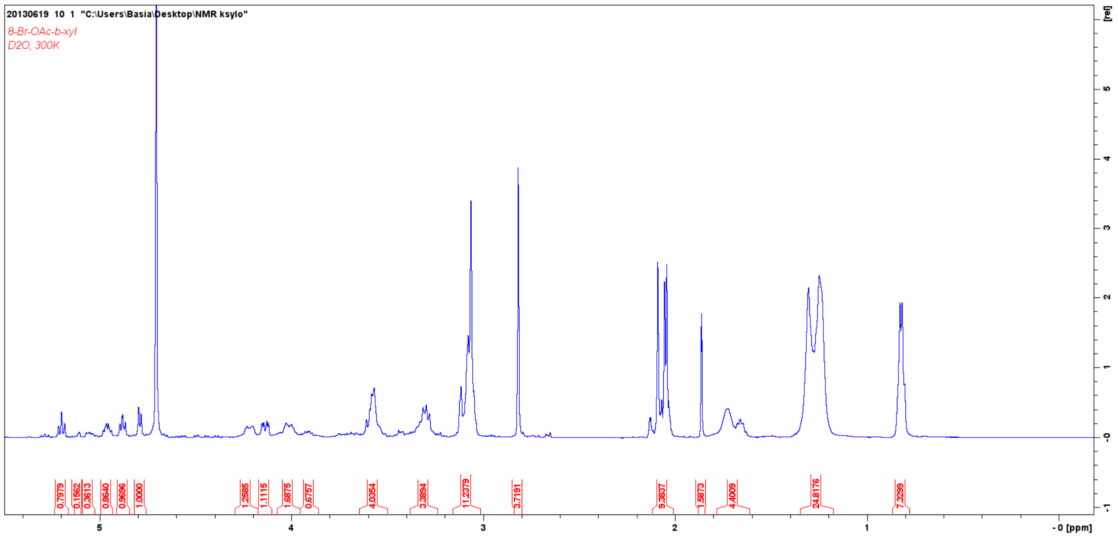

^{13}C NMR (125 MHz, D_2O):

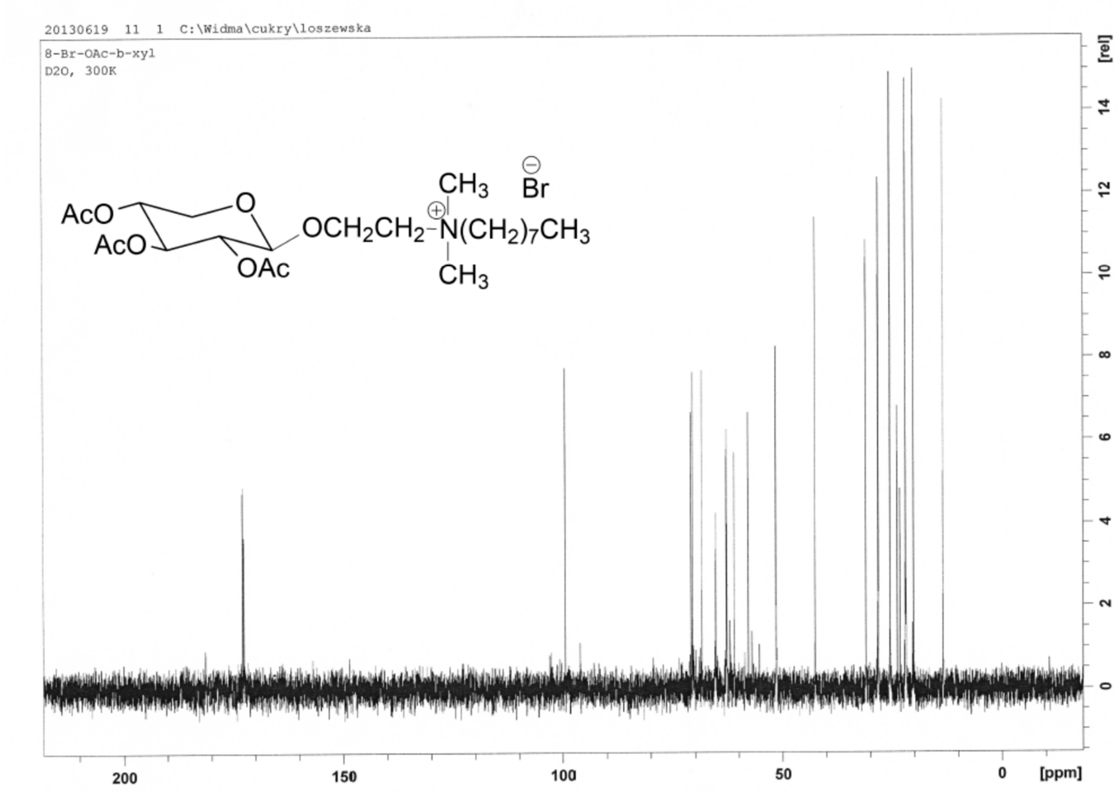

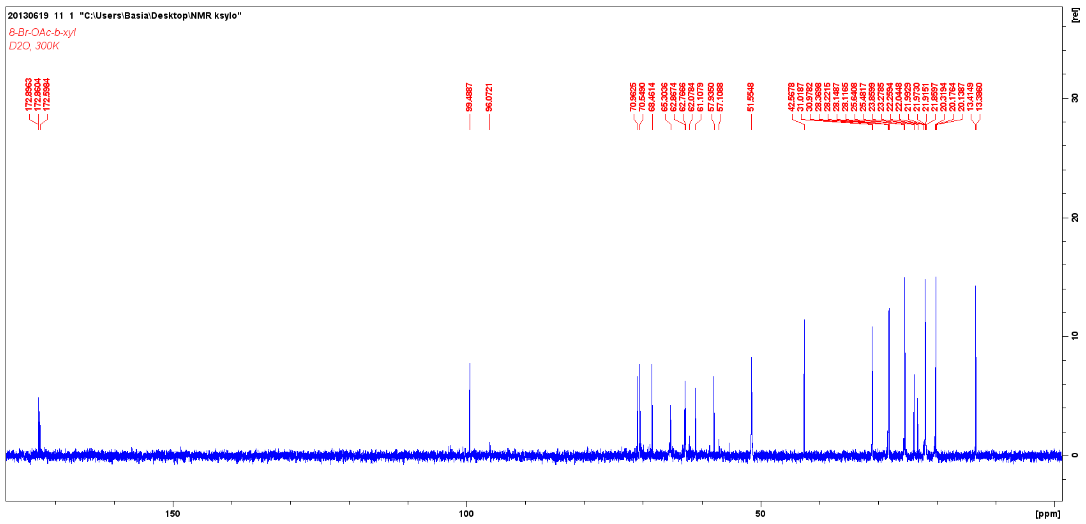

^{1}H-^{1}H COSY NMR:

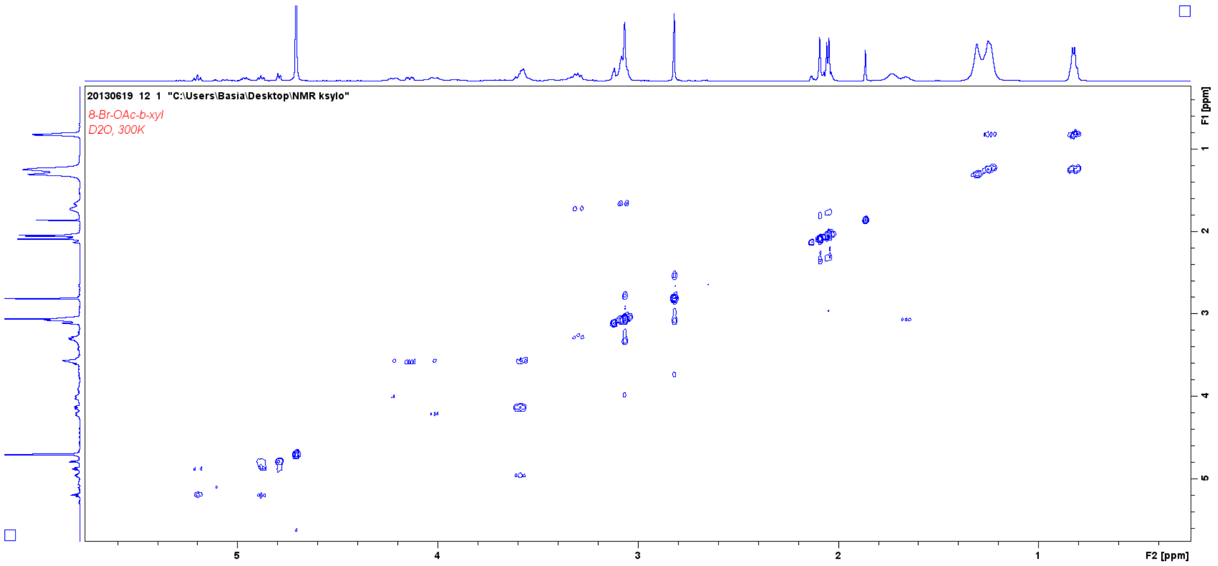

^{1}H-^{13}C HSQC NMR:

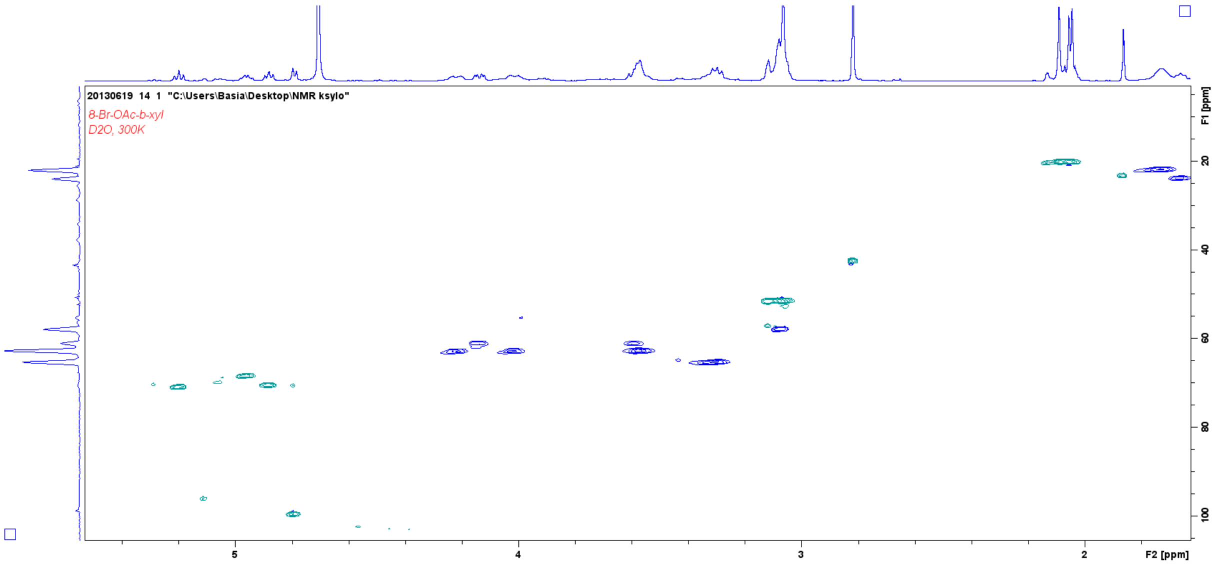

N-[2-(2′,3′,4′-tri-O-acetyl-α-D-xylopyranosyloxy)ethyl]pyridinium bromide (**5a**)

^{1}H NMR (500 MHz, D_2O):

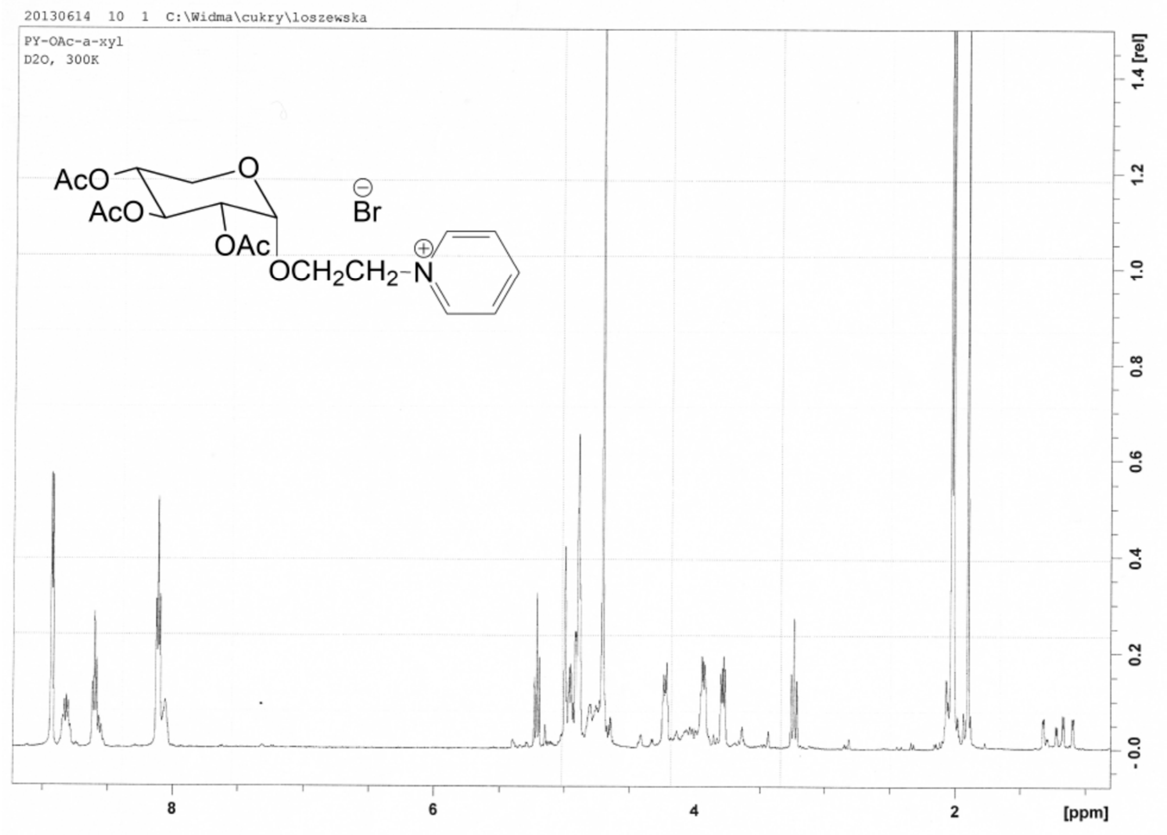

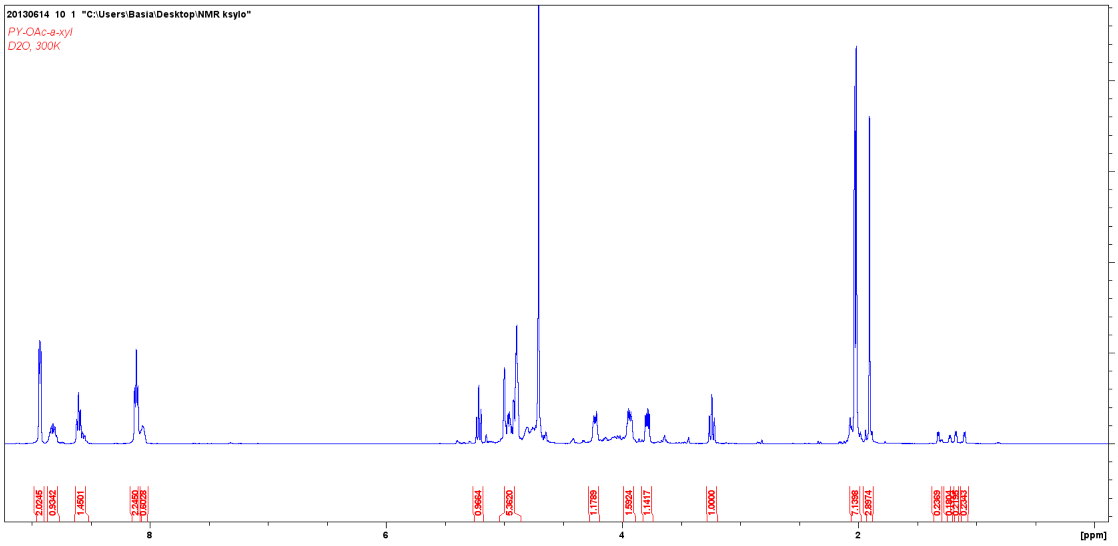

^{13}C NMR (125 MHz, D_2O):

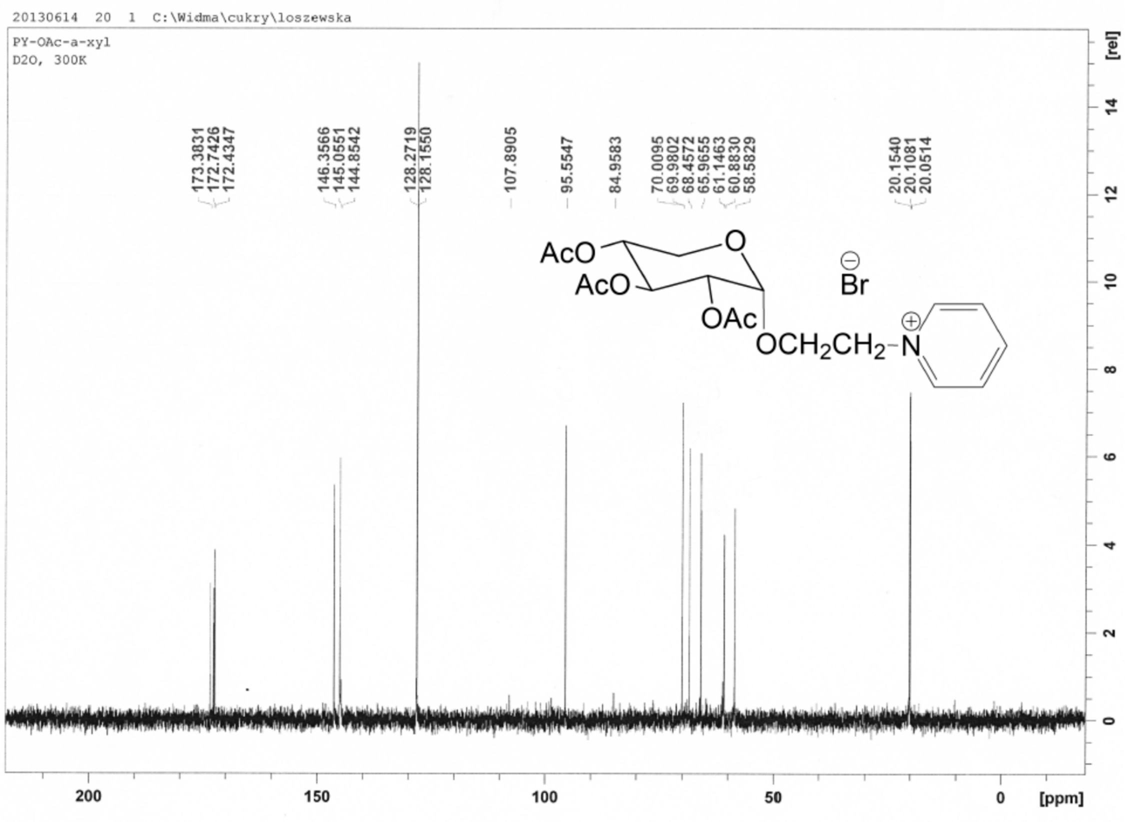

^{1}H-^{1}H COSY NMR:

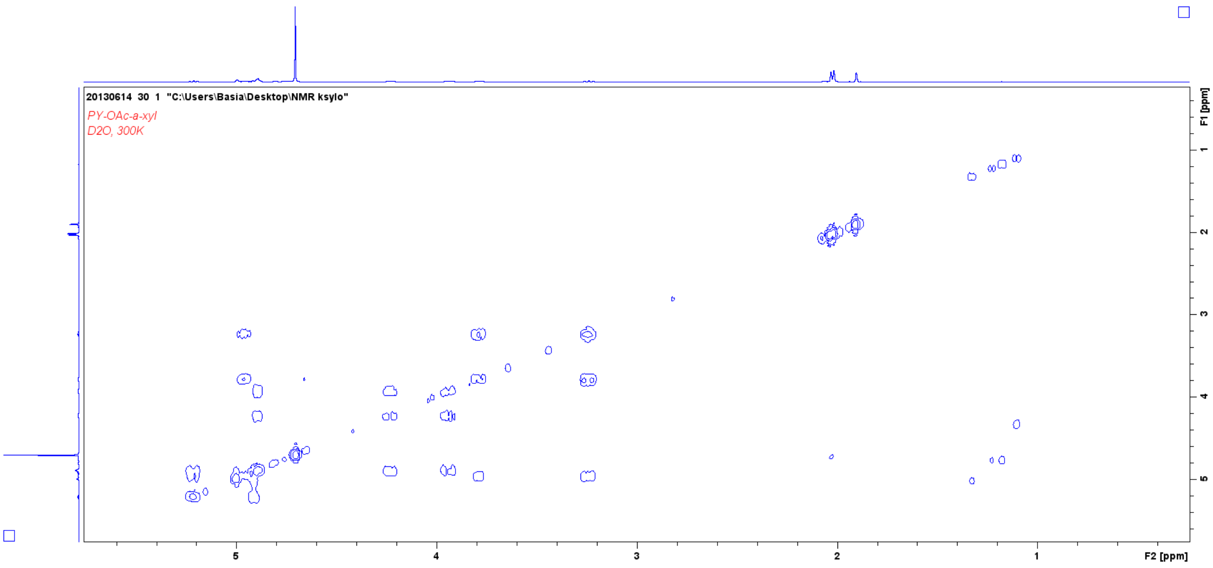

^{1}H-^{13}C HSQC NMR:

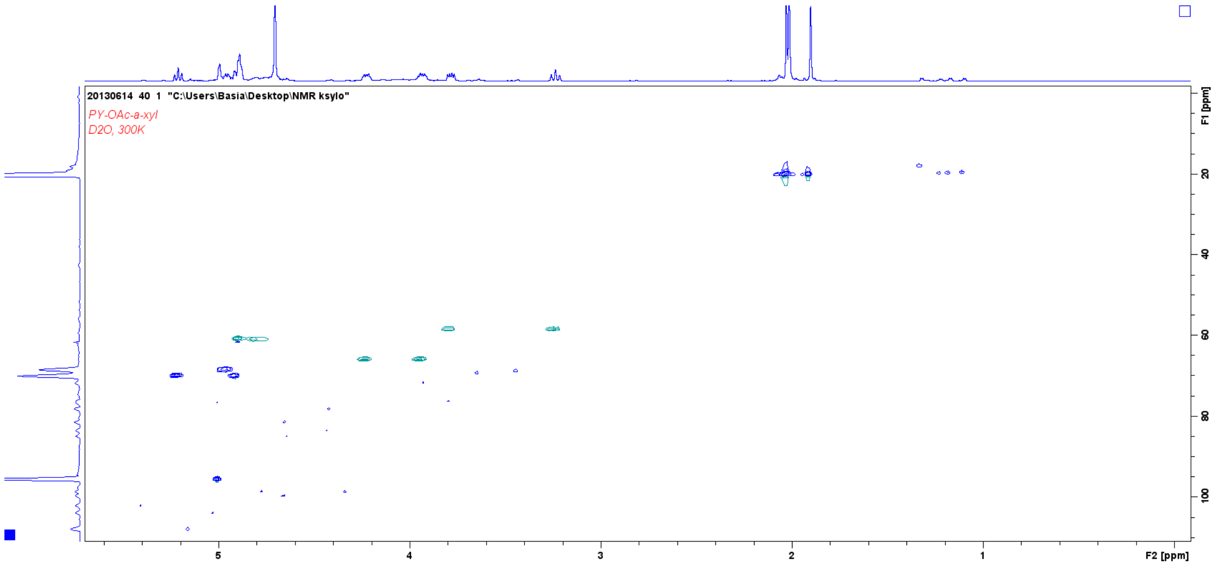

N-[2-(2′,3′,4′-tri-O-acetyl-*α*-D-xylopyranosyloxy)ethyl]]-*N*,*N*,*N*-trimethylammonium bromide (**5b**)

^{1}H NMR (500 MHz, D_2O):

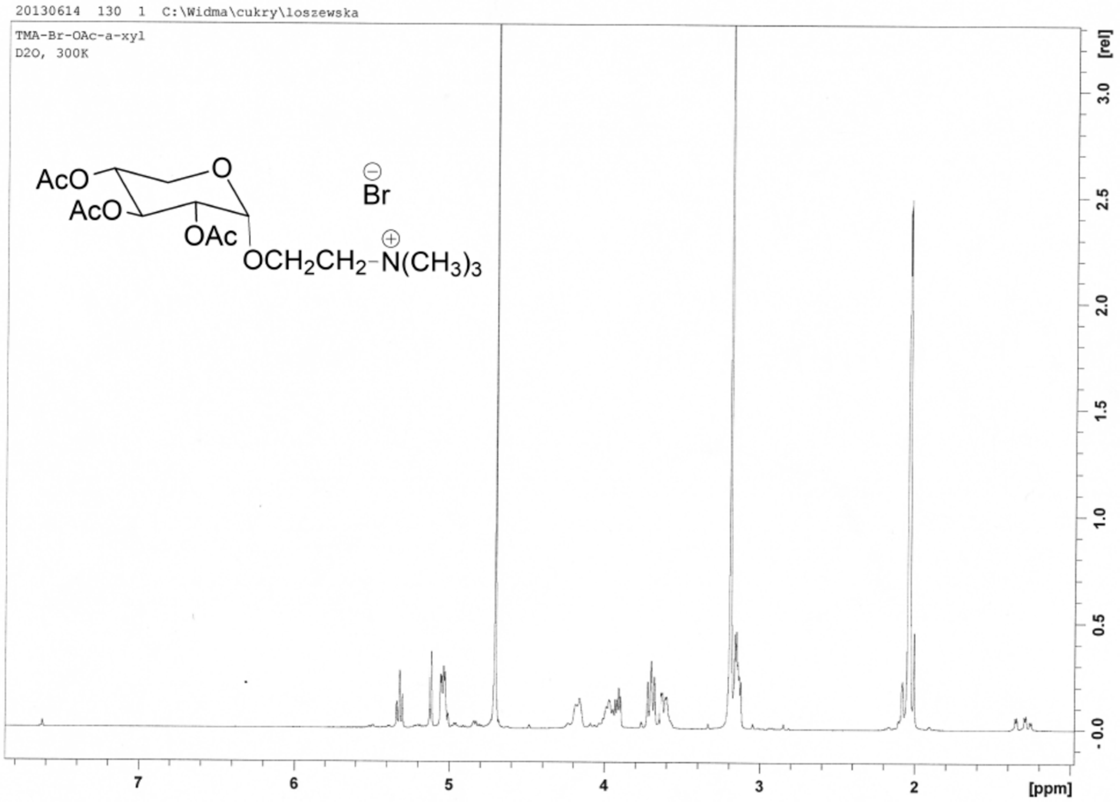

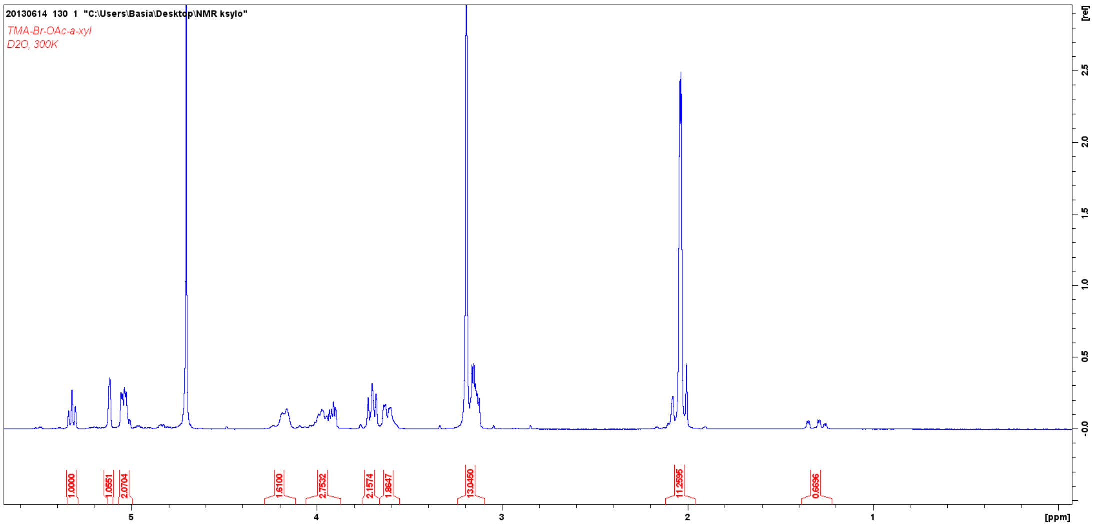

^{13}C NMR (125 MHz, D_2O):

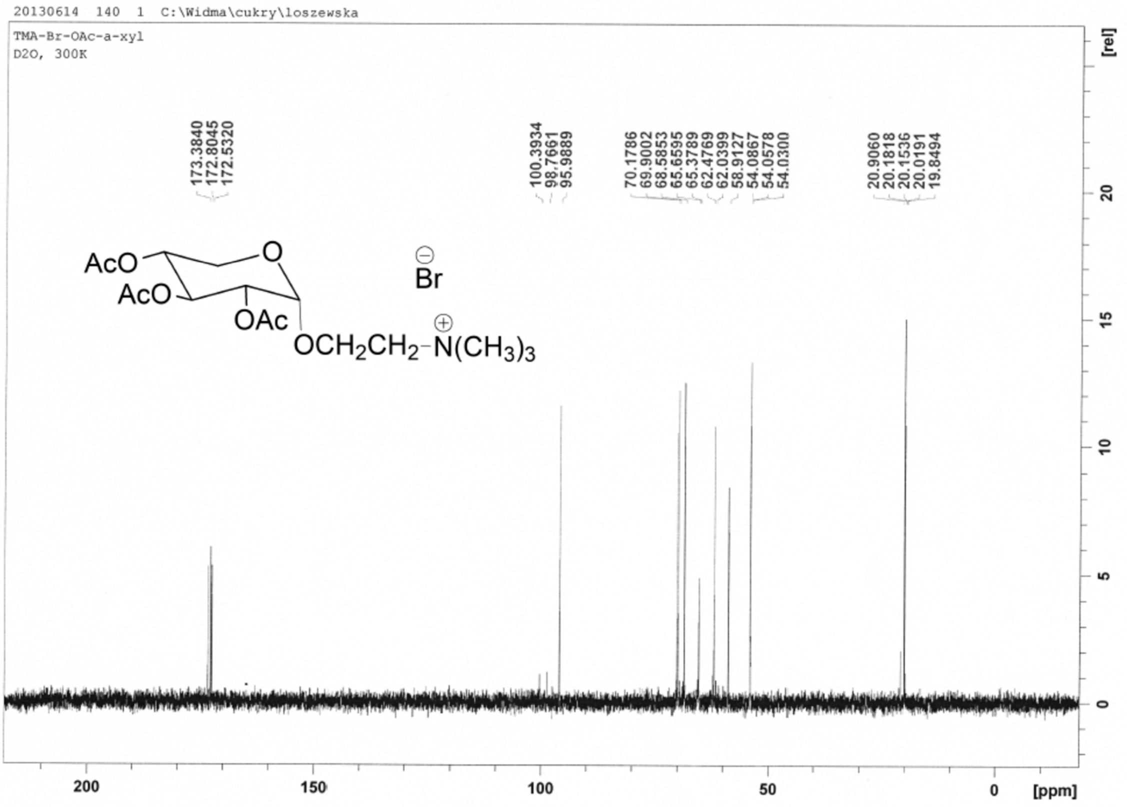

^{1}H-^{1}H COSY NMR:

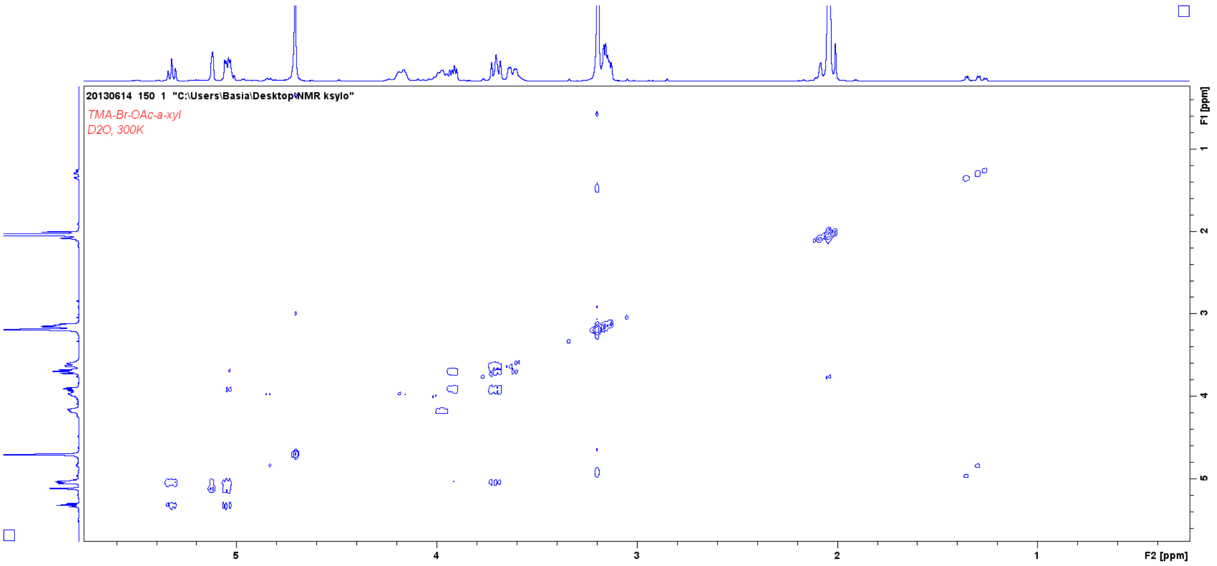

^{1}H-^{13}C HSQC NMR:

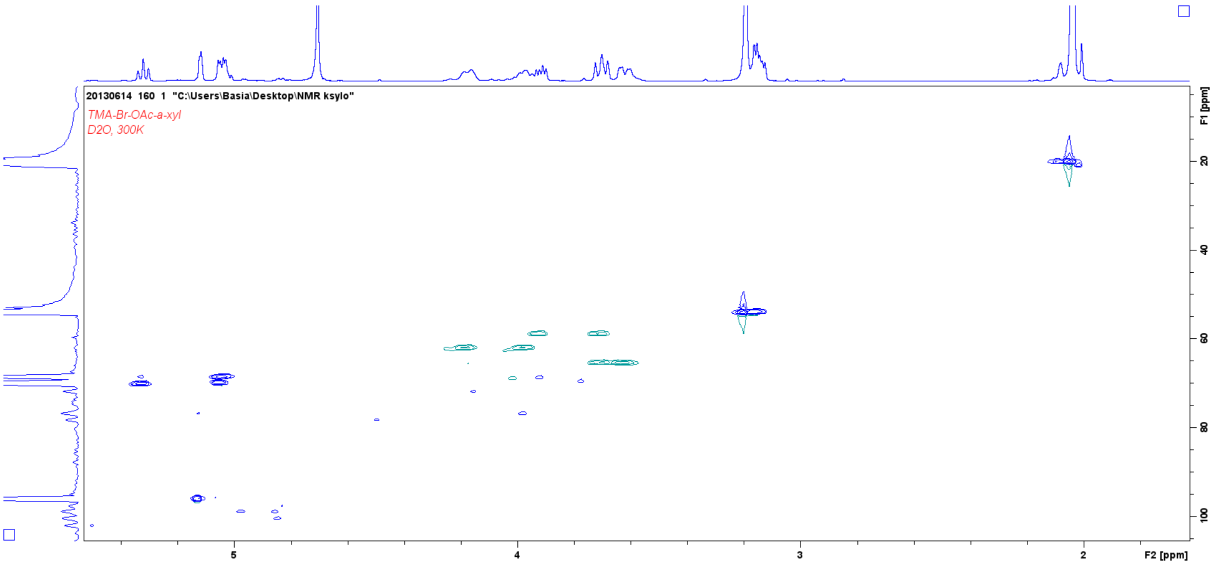

N-[2-(2′,3′,4′-tri-O-acetyl-*α*-D-xylopyranosyloxy)ethyl]-*N*-hexyl-*N*,*N*-dimethylammonium bromide (**5c**)

^{1}H NMR (500 MHz, D_2O):

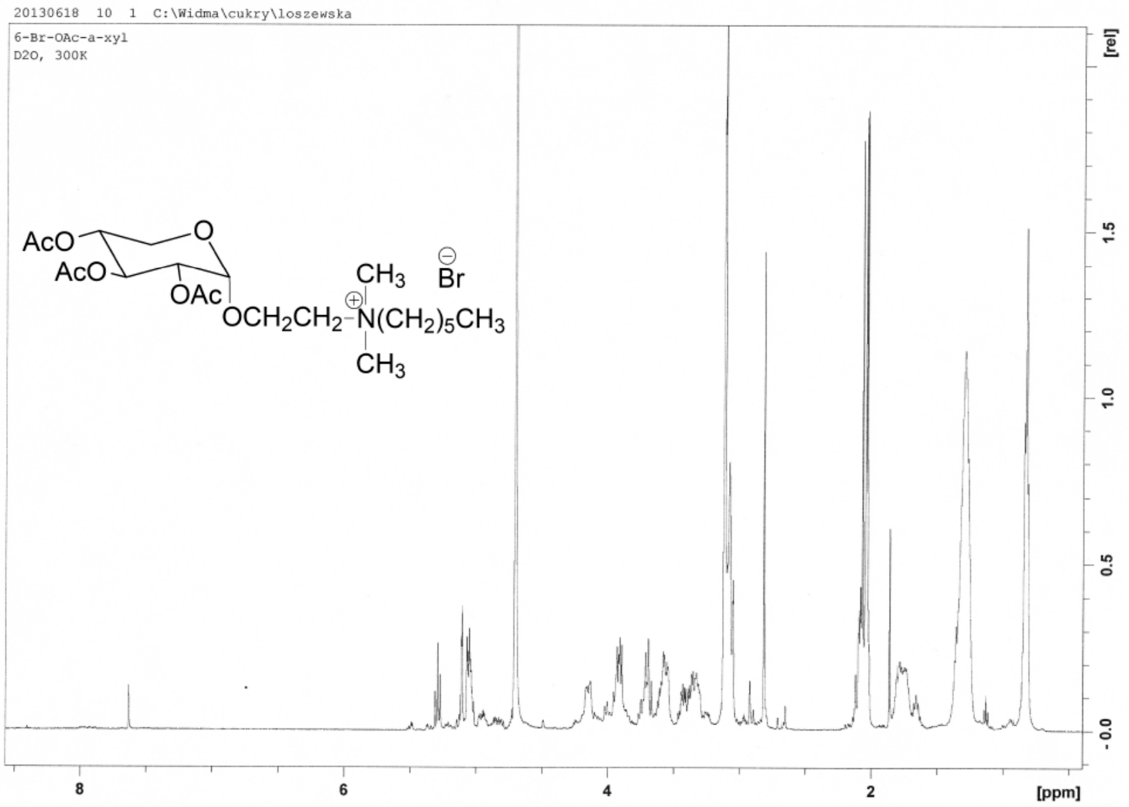

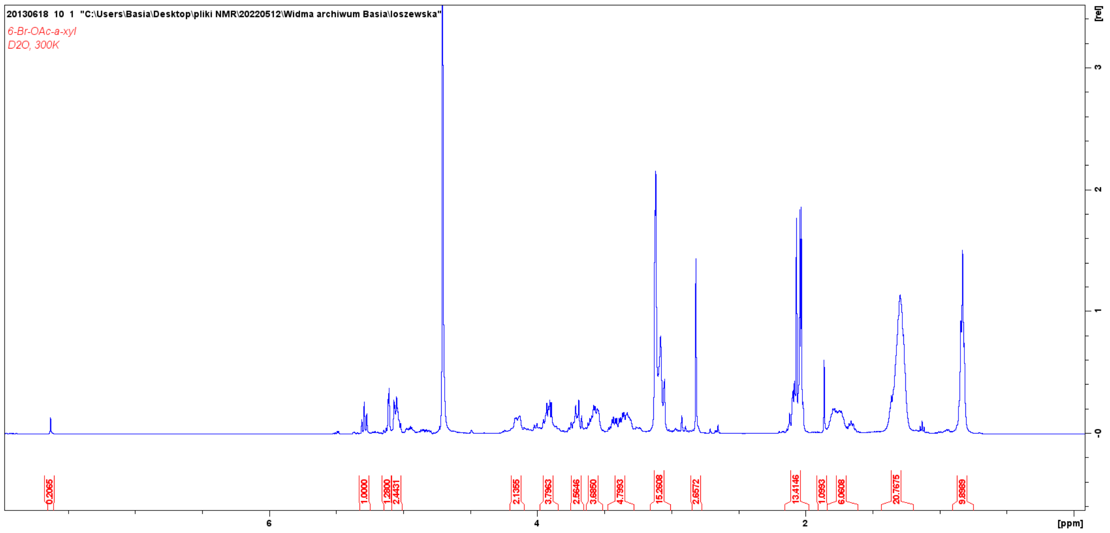

^{13}C NMR (125 MHz, D_2O):

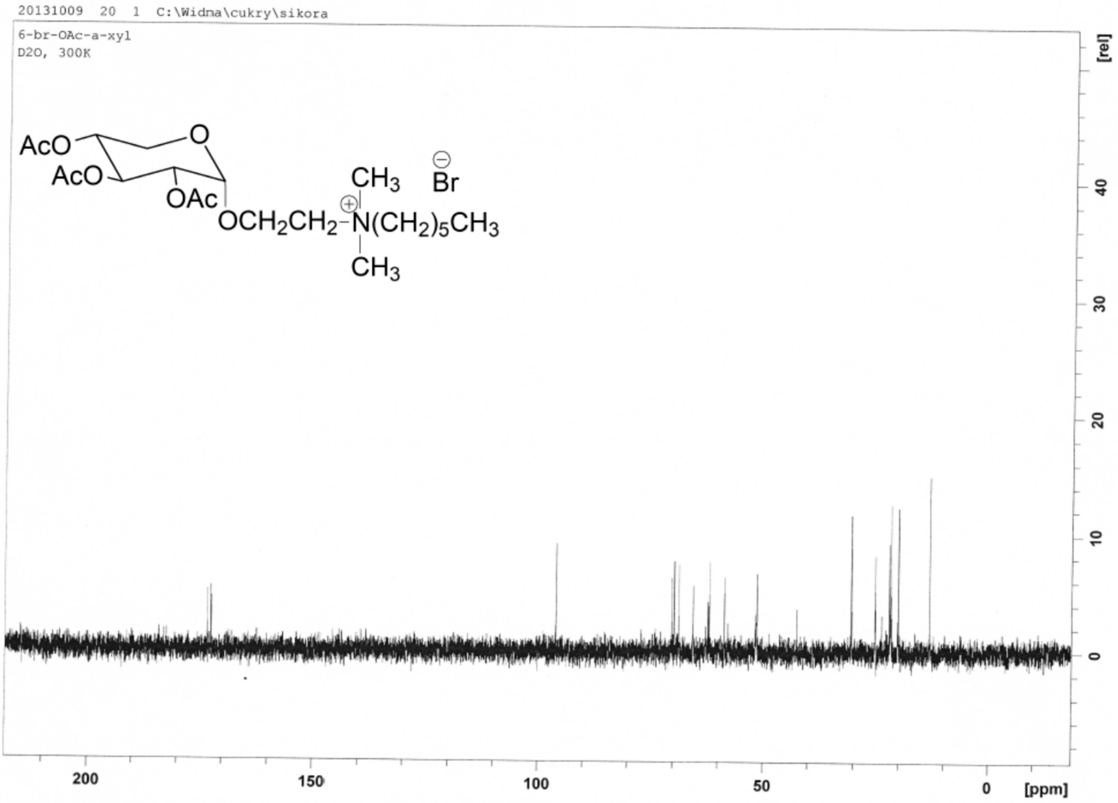

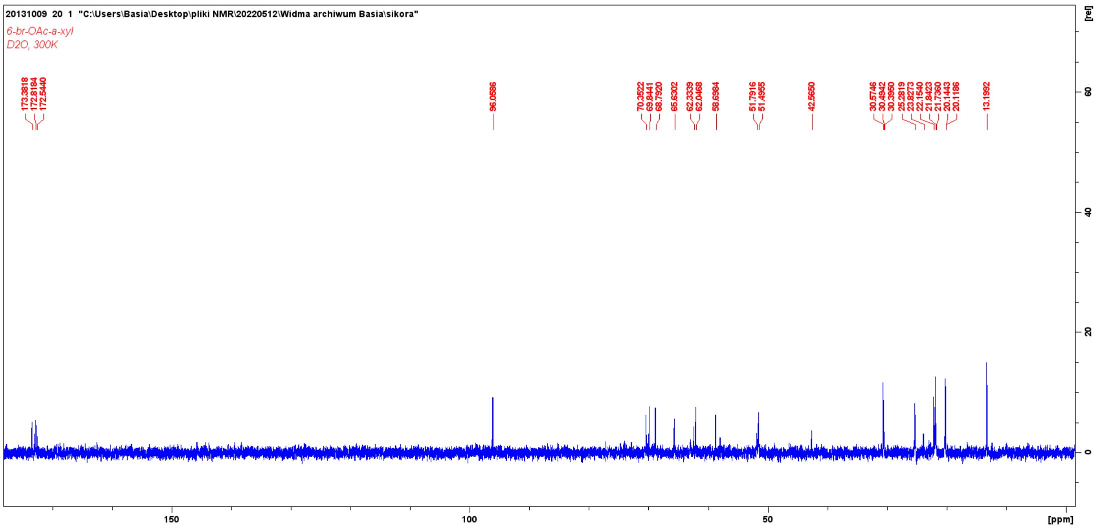

N-[2-(2′,3′,4′-tri-O-acetyl-α-D-xylopyranosyloxy)ethyl]-*N*,*N*-dimethyl-*N*-octylammonium bromide (**5d**)

^{1}H NMR (500 MHz, D_2O):

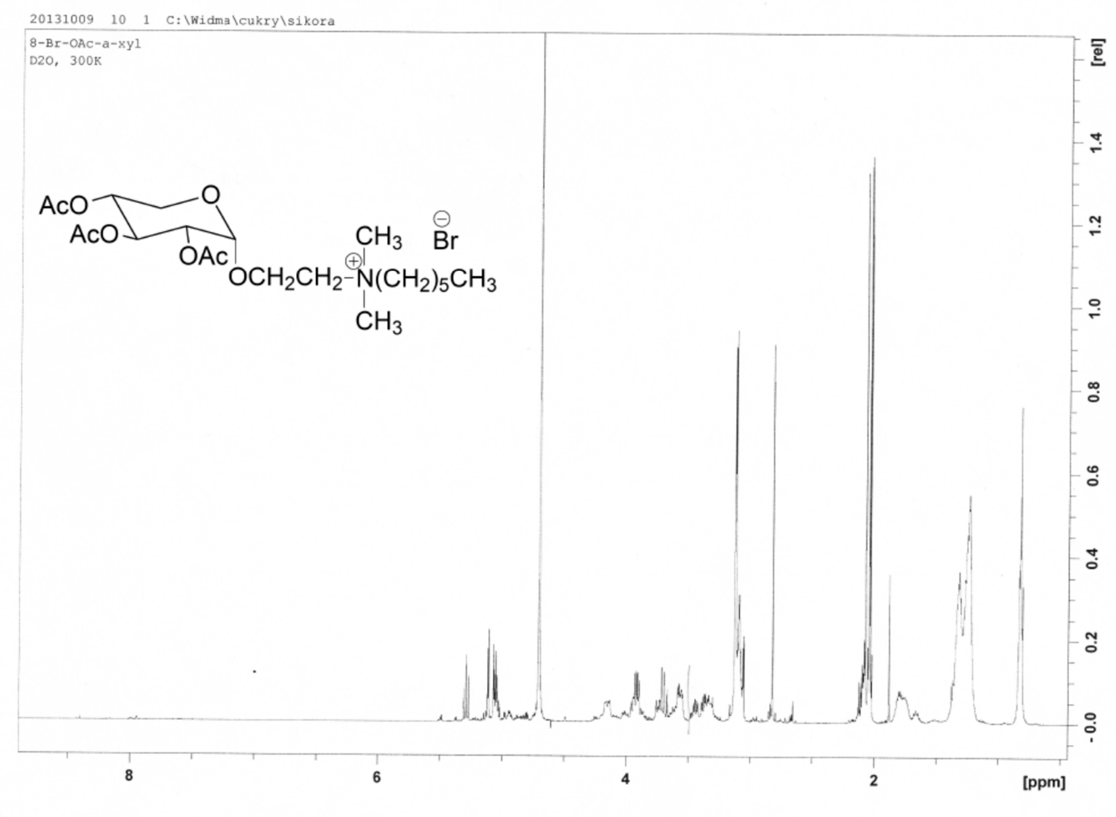

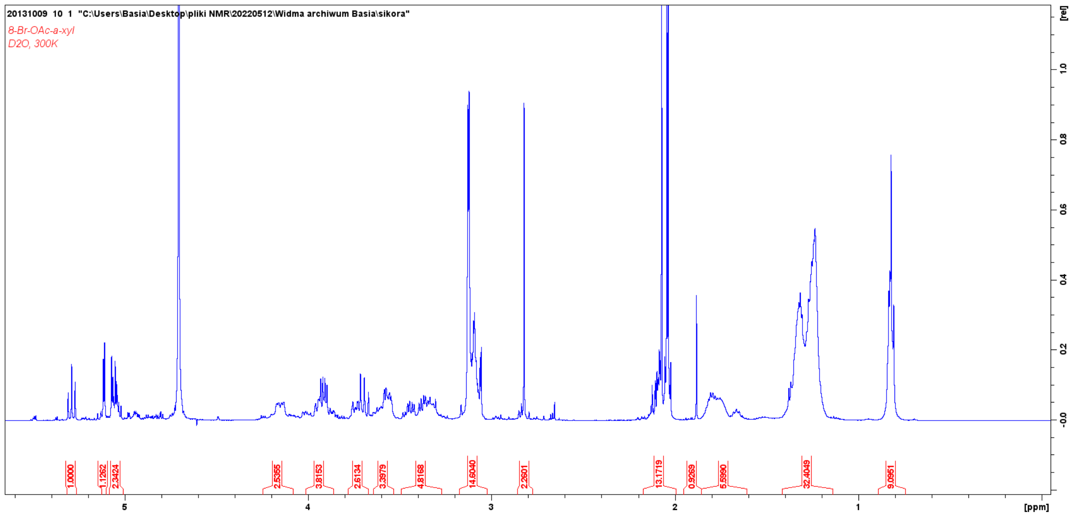

^{13}C NMR (125 MHz, D_2O):

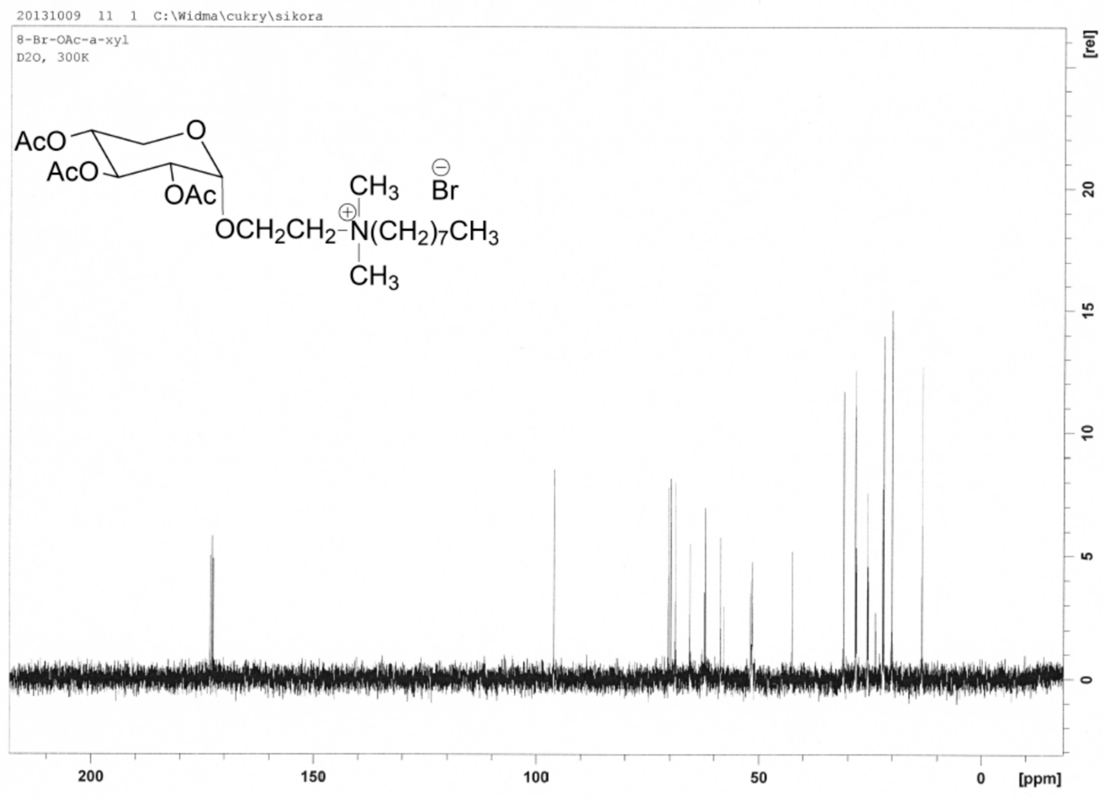

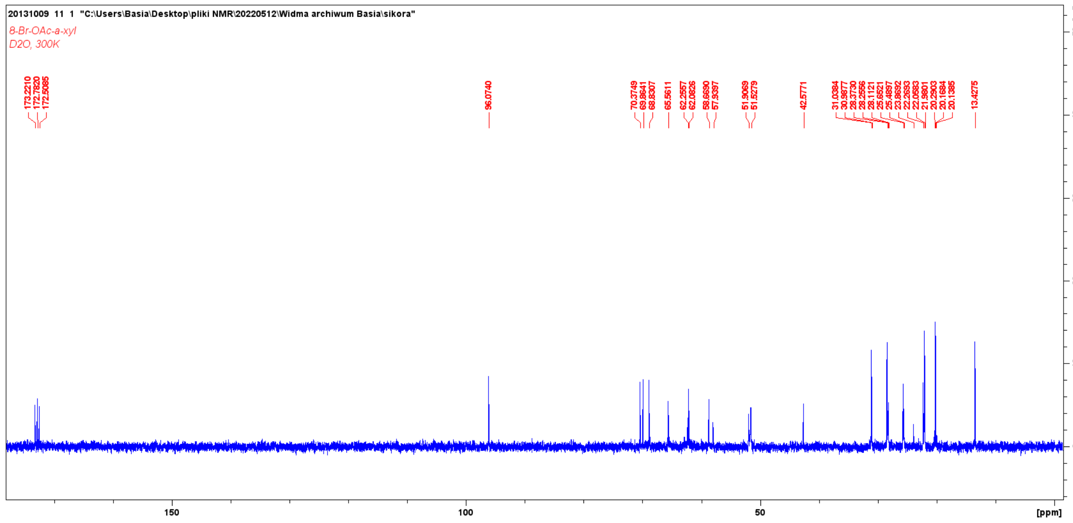

^{1}H-^{1}H COSY NMR:

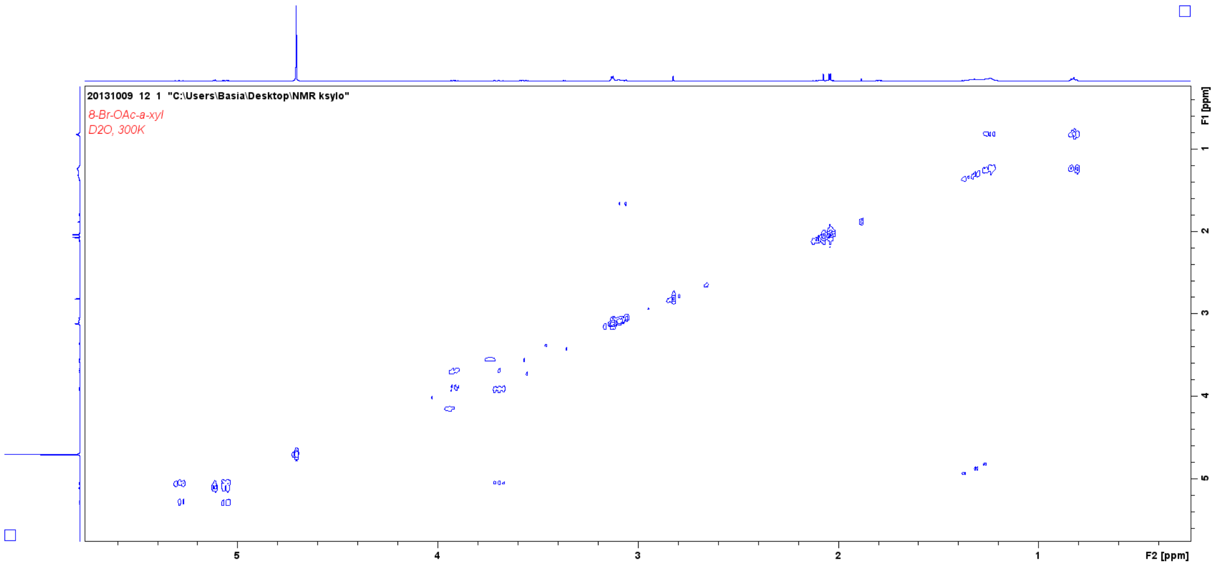

^{1}H-^{13}C HSQC NMR:

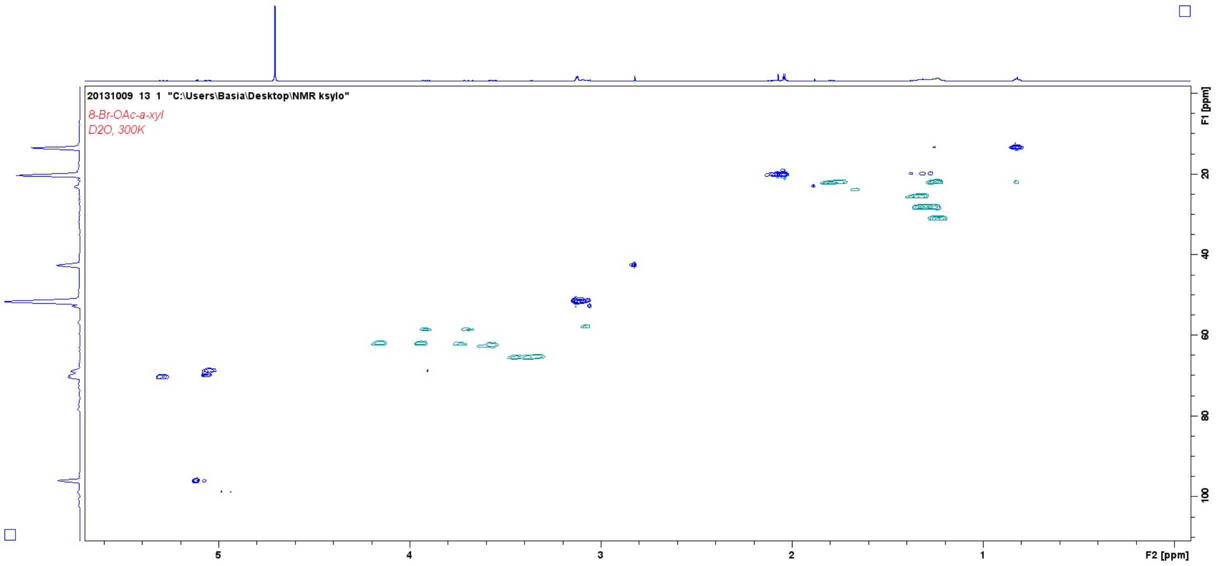

4. MS SPECTRA

N-[2-(2′,3′,4′-tri-O-acetyl-β-D-xylopyranosyloxy)ethyl]pyridinium bromide (**4a**) *m/z* 382.1492 ([M-Br]$^+$)

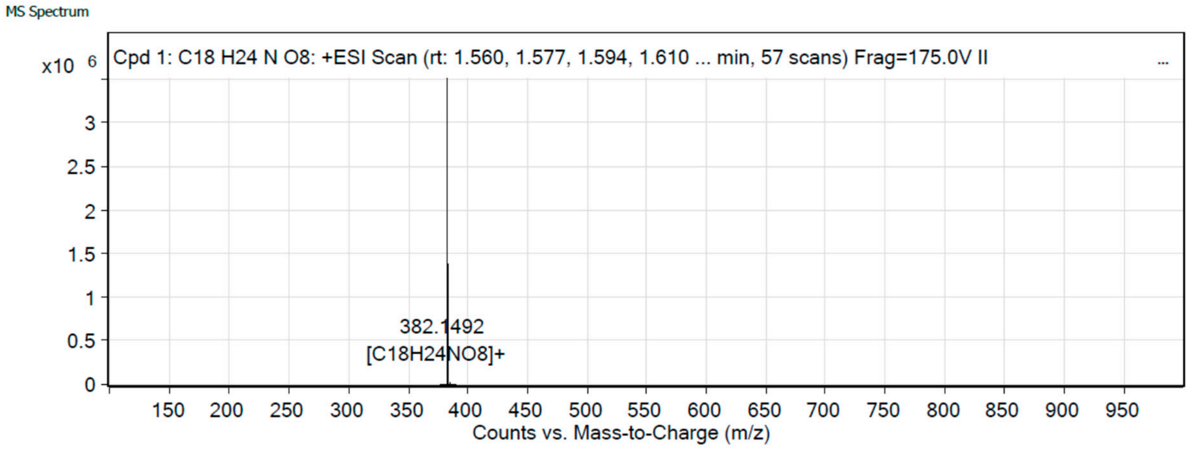

N-[2-(2′,3′,4′-tri-O-acetyl-β-D-xylopyranosyloxy)ethyl]]-*N*,*N*,*N*-trimethylammonium bromide (**4b**) *m/z* 362.1805 ([M-Br]$^+$)

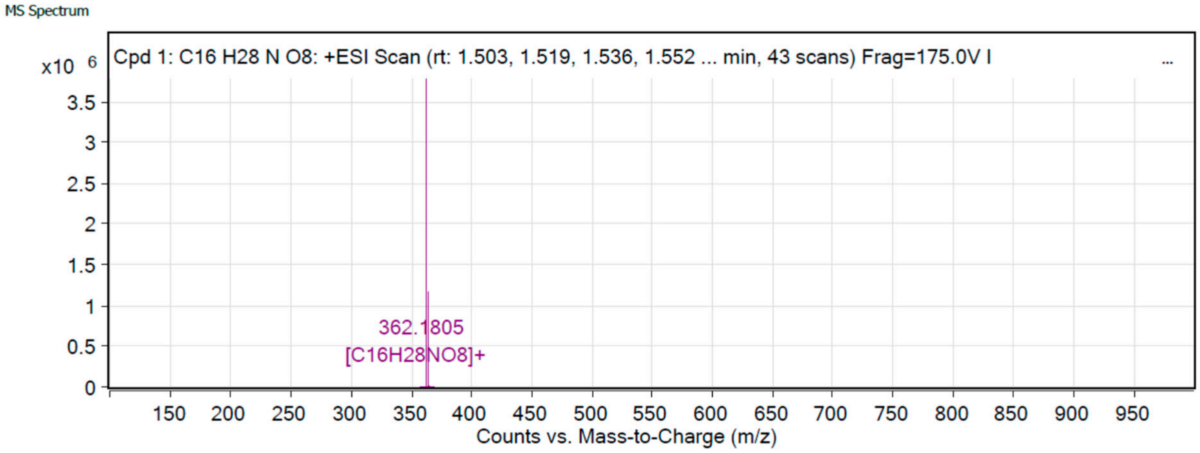

N-[2-(2′,3′,4′-tri-O-acetyl-β-D-xylopyranosyloxy)ethyl]-*N*-hexyl-*N*,*N*-dimethylammonium bromide (**4c**) *m/z* 432.2587 ([M-Br]$^+$)

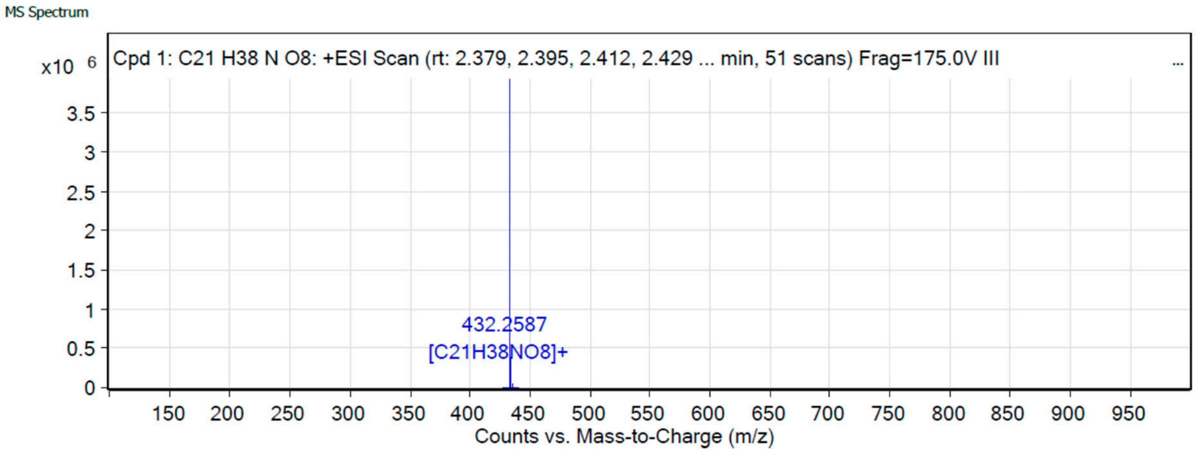

N-[2-(2′,3′,4′-tri-O-acetyl-β-D-xylopyranosyloxy)ethyl]-*N*,*N*-dimethyl-*N*-octylammonium bromide (**4d**) *m*/*z* 460.2898 ([M-Br]+)

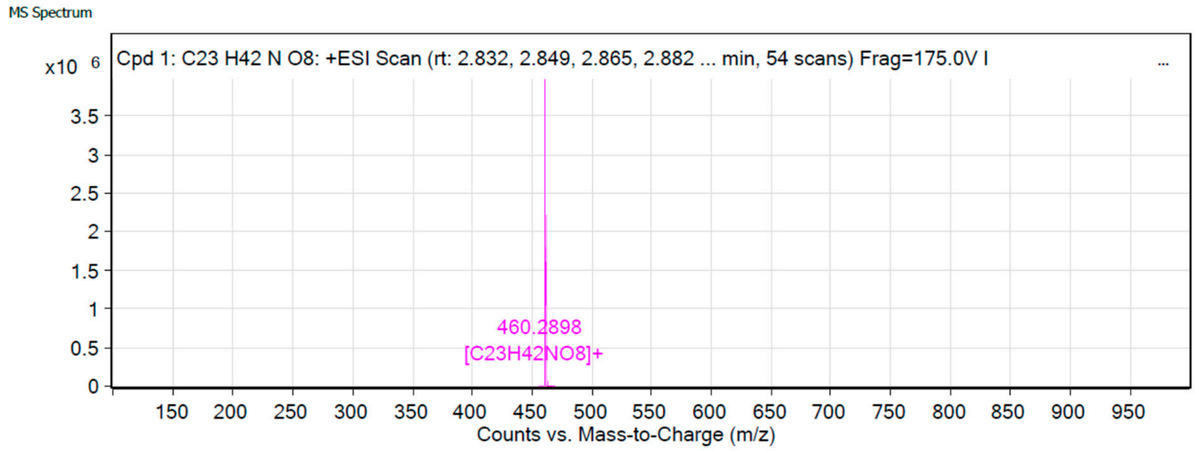

N-[2-(2′,3′,4′-tri-O-acetyl-α-D-xylopyranosyloxy)ethyl]pyridinium bromide (**5a**) *m*/*z* 382.1492 ([M-Br]+)

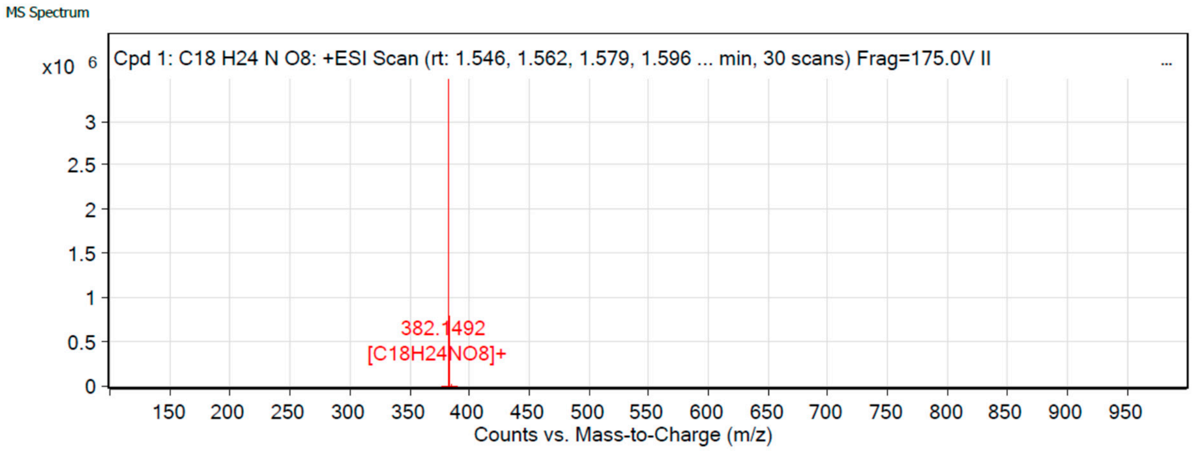

N-[2-(2′,3′,4′-tri-O-acetyl-α-D-xylopyranosyloxy)ethyl]]-*N*,*N*,*N*-trimethylammonium bromide (**5b**) *m*/*z* 362.1804 ([M-Br]+)

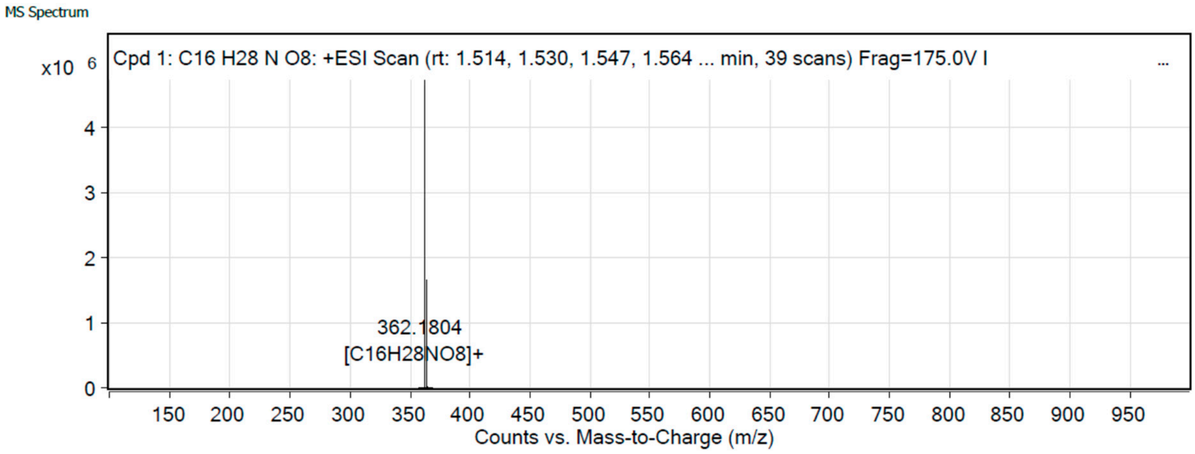

N-[2-(2′,3′,4′-tri-O-acetyl-α-D-xylopyranosyloxy)ethyl]-*N*-hexyl-*N*,*N*-dimethylammonium bromide (**5c**) *m*/*z* 432.2587 ([M-Br]+)

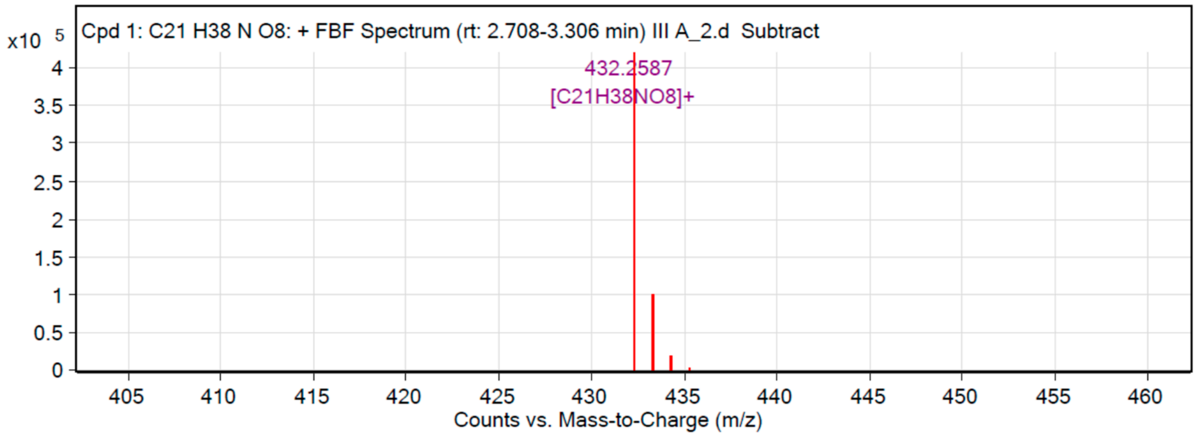

N-[2-(2′,3′,4′-tri-O-acetyl-α-D-xylopyranosyloxy)ethyl]-*N*,*N*-dimethyl-*N*-octylammonium bromide (**5d**) *m*/*z* 460.2899 ([M-Br]+)

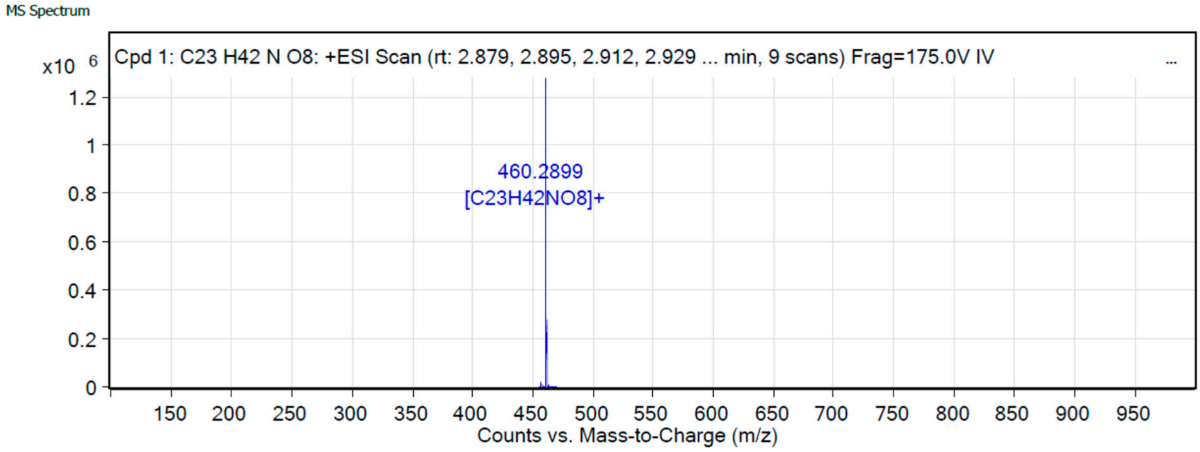

References

1. Clinical and Laboratory Standards Institute (CLSI). *Methods for Dilution Antimicrobial Susceptibility Tests for Bacteria That Grow Aerobically; Approved Standard*, 9th ed.; CLSI: Berwyn, PA, USA, 2012; pp.:32.
2. Clinical Laboratory Standards Institute. Reference Method for Broth Dilution Antifungal susceptibility testing of yeast. *Clin. Lab. Stand. Inst.* **2012**, *32*, 1–23.
3. Maron, D.M.; Ames, B.N. Revised methods for the Salmonella mutagenicity test. *Mutat. Res. Mutagen. Relat. Subj.* **1983**, *113*, 173–215, doi:10.1016/0165-1161(83)90010-9.
4. Mortelmans, K.; Zeiger, E. The Ames Salmonella/microsome mutagenicity assay. *Mutat. Res. Mol. Mech. Mutagen.* **2000**, 455, 29–60, doi:10.1016/S0027-5107(00)00064-6.
5. Gulluce, M.; Agar, G.; Baris, O.; Karadayi, M.; Orhan, F.; Sahin, F. Mutagenic and antimutagenic effects of hexane extract of some Astragalus species grown in the eastern Anatolia region of Turkey. *Phyther. Res.* **2010**, *24*, 1014–1018, doi:10.1002/ptr.3059.
6. Biso, F.I.; Rodrigues, C.M.; Rinaldo, D.; Reis, M.B. dos; Bernardi, C.C.; de Mattos, J.C.P.; Caldeira-de-Araújo, A.; Vilegas, W.; Cólus, I.M. de S.; Varanda, E.A. Assessment of DNA damage induced by extracts, fractions and isolated compounds of Davilla nitida and Davilla elliptica (Dilleniaceae). *Mutat. Res. Toxicol. Environ. Mutagen.* **2010**, *702*, 92–99, doi:10.1016/j.mrgentox.2010.07.011.
